# Supplementary material for: IndEcho study: cohort study investigating birth size, childhood growth and young adult cardiovascular risk factors as predictors of midlife myocardial structure and function in South Asians
Source: BMJ Open. 2018 Apr 10;8(4):e019675. doi: 10.1136/bmjopen-2017-019675 (PMC5898335; doi:10.1136/bmjopen-2017-019675)
Supplement: Supplementary file 1 [file bmjopen-2017-019675supp001.pdf]

Supplementary table 1: Total number of participants in Phase 1 and 2 of NDBC and VBC and reasons for lost to follow-up between both adult phases.

	NDBC	VBC
**Phase-1**	(1998-2002)	(1998-2002)
Age (years)	29.3 ± 1.0	28.3 ± 1.2
Total (N)	1,526	2,218
Men (N)	886	1,161
Women (N)	640	1,057
**Phase-2**	(2006-2009)	(2013-2014)
Age (years)	36.1 ± 1.1	41.6 ± 1.0
Total (N)	1,100	1,080
Men (N)	665	581
Women (N)	484	499
**Numbers lost to follow-up between Phase 1 and Phase 2 and reasons**	426	1,138
Known deaths (N)	18	62
Known migration (N)	88	50
Unwilling to participate (N)	266	49
Not traceable (N)	54	21
Funding restrictions (N)	0	956

Supplementary table 2: Co-variates that will be studied to assess lifestyle and behavior in the IndEcho participants

Exposure variable	Questionnaire	Variables included in the questionnaire
Socioeconomic status (SES)[51-53]	The Government of India National Family Health Survey (NFHS-2) questionnaire and Standard of Living Index (SLI – IIPS,2000) questionnaire that contains 11 items to measure SES for both rural and urban populations of the entire country	Includes information on:<br>(i) Education<br>(ii) Occupation<br>(iii) Household characteristics (family type, number of persons)<br>(iv) SLI is a summary household measure composed of 11 items, including (a) house type (b) source of lighting (c) toilet facilities (d) main fuel for cooking (e) source of drinking water (f) separate room for cooking (g) ownership of house (h) ownership of agricultural land (i) ownership of irrigated land (j) ownership of livestock (k) ownership of durable goods
Physical activity (PA)[54,55]	Global Physical Activity Questionnaire (GPAQ)	Includes questions related to three domains<br>(i) Activity at work and in leisure time (vigorous- and moderate-intensity activity)<br>(ii) Travel to and from places<br>(iii) Recreational activities (vigorous and moderate recreation) and sitting behavior
Smoking and alcohol consumption[56]	NFHS-2 Questionnaire	Duration and quantity/type of tobacco consumed, smoking habits and alcohol intake
