# Supplementary material for: IndEcho study: cohort study investigating birth size, childhood growth and young adult cardiovascular risk factors as predictors of midlife myocardial structure and function in South Asians
Source: BMJ Open. 2018 Apr 10;8(4):e019675. doi: 10.1136/bmjopen-2017-019675 (PMC5898335; doi:10.1136/bmjopen-2017-019675)
Supplement: Author's manuscript [file bmjopen-2017-019675.draft_revisions.pdf]

BMJ Open is committed to open peer review. As part of this commitment we make the peer review history of every article we publish publicly available.

When an article is published we post the peer reviewers' comments and the authors' responses online. We also post the versions of the paper that were used during peer review. These are the versions that the peer review comments apply to.

The versions of the paper that follow are the versions that were submitted during the peer review process. They are not the versions of record or the final published versions. They should not be cited or distributed as the published version of this manuscript.

BMJ Open is an open access journal and the full, final, typeset and author-corrected version of record of the manuscript is available on our site with no access controls, subscription charges or pay-per-view fees (http://bmjopen.bmj.com).

If you have any questions on BMJ Open's open peer review process please email info.bmjopen@bmj.com

BMJ Open

The IndEcho study: Cohort study investigating birth size, childhood growth and young adult cardiovascular risk factors as predictors of mid-life myocardial structure and function in South Asians

Journal:	*BMJ Open*
Manuscript ID	bmjopen-2017-019675
Article Type:	Protocol
Date Submitted by the Author:	19-Sep-2017
Complete List of Authors:	Vasan, Senthil; University of Oxford, Radcliffe Department of Medicine; University of Southampton, MRC Lifecourse Epidemiology Unit<br>Roy, Ambuj; All India Institute of Medical Sciences, Cardiology<br>Samuel, Viji; Christian Medical College and Hospital Vellore, Cardiology<br>Antonisamy, Belavendra; Christian Medical College and Hospital Vellore, Biostatistics<br>Bhargava, Santosh; Sunder Lal Jain Hospital<br>Alex, Anoop; Christian Medical College and Hospital Vellore, Cardiology<br>Singh, Bhaskar; Sunder Lal Jain Hospital<br>Osmond, Clive; University of Southampton , MRC Lifecourse Epidemiology Unit<br>Geethanjali, Finney; Christian Medical College and Hospital Vellore, Clinical Biochemistry<br>Karpe, Fredrik; University of Oxford , Oxford center for Diabetes, Endocrinology and Metabolism<br>Sachdev, Harshpal; Sitaram Bhartia Institute of Science and Research, Department of Paediatrics<br>Agrawal, Kanhaiya ; Christian Medical College and Hospital Vellore, Endocrinology<br>Ramakrishnan, Lakshmy; All India Institute of Medical Sciences,<br>Tandon, Nikhil; All India Institute of Medical Sciences,<br>THOMAS, NIHAL; CHRISTIAN MEDICAL COLLEGE, ENDOCRINOLOGY<br>Premkumar, Prasanna; Christian Medical College and Hospital Vellore, Biostatistics<br>Asaithambi, Prrathepa ; Christian Medical College and Hospital Vellore, Endocrinology<br>Princy , Sneha; Christian Medical College and Hospital Vellore, Biostatistics<br>Sinha , Sikha; Sitaram Bhartia Institute of Science and Research<br>PAUL, THOMAS; CHRISTIAN MEDICAL COLLEGE, ENDOCRINOLOGY&DIABETES<br>Prabhakaran, Dorairaj; Centre for Chronic Disease Control, ; Public Health Foundation of India,<br>Fall, Caroline ; University of Southampton, MRC Lifecourse Epidemiology Unit
**Primary Subject Heading**:	Cardiovascular medicine
Secondary Subject Heading:	Diabetes and endocrinology, Epidemiology, Global health

Keywords:	IndEcho, South Asians, Echocardiography < CARDIOLOGY, Adiposity, Left ventricular mass and function, Birth weight and early growth

SCHOLARONE™
Manuscripts

For peer review only

The IndEcho study: Cohort study investigating birth size, childhood growth and young adult cardiovascular risk factors as predictors of mid-life myocardial structure and function in South Asians

Senthil K Vasan[1,2], Ambuj Roy[3,4], Viji Thomson Samuel[5], Belavendra Antonisamy[5], Santosh K Bhargava[6], Anoop George Alex[5], Bhaskar Singh[6], Clive Osmond[1], Finney S Geethanjali[5], Fredrik Karpe[2], Harshpal Sachdev[7], Kanhaiya Agrawal[5], Lakshmy Ramakrishnan[4], Nikhil Tandon[4], Nihal Jacob Thomas[5], Prasanna S Premkumar[5], Prrathepa Asaithambi[5], Sneha F.X. Princy[5], Sikha Sinha[7], Thomas Vizhalil Paul[5], Dorairaj Prabhakaran[3,8], Caroline HD Fall[1]

[1]MRC Lifecourse Epidemiology Unit, University of Southampton, Southampton General Hospital, Southampton UK
[2]Oxford center for Diabetes, Endocrinology and Metabolism, Radcliffe Department of Medicine, University of Oxford, Oxford, UK
[3]Centre for Chronic Disease Control, Gurgaon, New Delhi, India
[4]All-India Institute of Medical Sciences, New Delhi, India
[5]Christian Medical College, Vellore, India
[6]Sunder Lal Jain Hospital, New Delhi, India
[7]Sitaram Bhartia Institute of Science and Research, New Delhi, India
[8]Public Health Foundation of India, Gurgaon, India

For peer review only

Key words: IndEcho, South Asians, Echocardiography, Left ventricular mass, Left ventricular function, Adiposity, Cardiovascular disease risk markers, Birth weight, Child growth

Address for correspondence:

Caroline HD Fall

MRC Lifecourse Epidemiology Unit

University of Southampton

Southampton General Hospital

Tremona Road, Southampton, Hampshire, UK

SO16 6YD

Tel: +44 2380 777624

Fax: +44 2380 704021

Email: chdf@mrc.soton.ac.uk

Word count: Abstract. 299/300; Text 3492/4000 words

For peer review only - http://bmjopen.bmj.com/site/about/guidelines.xhtml

ABSTRACT

Introduction South Asians have high rates of cardiovascular disease (CVD) and its risk factors (hypertension, diabetes, dyslipidaemia and central obesity). Left ventricular (LV) hypertrophy and dysfunction are features of these disorders and important predictors of CVD mortality. Lower birth and infant weight, and greater childhood weight gain are associated with increased adult CVD mortality but there is little data on their relationship to LV function. The IndEcho study will examine associations of birth size, growth during infancy, childhood and adolescence, and CVD risk factors in young adulthood with mid-life cardiac structure and function in South Asian Indians.

Methods and analysis We propose to study approximately 3,000 men and women aged 43-50 years from two birth cohorts established in 1969-1973: the New Delhi Birth Cohort (NDBC; target N=1,526) and Vellore Birth Cohort (VBC; N=2,218). They had serial measurements of weight and height from birth to early adulthood. CVD risk markers (body composition, blood pressure, glucose tolerance and lipids) and lifestyle characteristics (tobacco and alcohol consumption, physical activity, socio-economic status) were assessed at age ~30 years. Clinical measurements in IndEcho will include anthropometry, blood pressure, biochemistry (glucose, fasting insulin and lipids, urinary albumin/creatinine ratio) and body composition by dual energy X-ray absorptiometry and bioelectrical impedance. Outcomes are LV mass and indices of LV systolic and diastolic function assessed by 2-D and Doppler echocardiography, carotid intima media thickness and electrocardiographic indicators of ischaemia. Regression and conditional growth models, adjusted for potential confounders, will be used to study associations of childhood and young adult exposures with these cardiovascular outcomes.

Ethics and dissemination The study has been approved by the Health Ministry Steering Committee, Government of India, and institutional ethics committees of participating centres in India, and the University of Southampton, UK. Results will be disseminated through scientific meetings and peer-reviewed journals.

Registration ISRCTN13432279.

For peer review only

Strengths and limitations of the study

- The study will provide the first longitudinal data linking size at birth, childhood growth and cardiovascular disease risk markers in young adult life with myocardial structure and function in mid-life in South Asians.
- It will include participants from rural and urban populations in north and south India, using harmonised protocols.
- A limitation of the study is attrition of the cohorts due to deaths and migration (mainly in childhood).
- Because this is a field-based study, echocardiography will be used, rather than cardiac MRI, which is the current gold standard method for measuring myocardial structure.

For peer review only

INTRODUCTION

Background and Rationale

The emerging epidemic of cardiovascular disease (CVD) in transitioning populations means that low and middle-income countries (LMICs) contribute a larger proportion to the global burden of CVD (~8-9 million deaths per year) than high income countries (~5 million).[1-3] Migrants from LMICs to high income countries experience an excess of CVD compared with indigenous populations.[4-6] A parallel increase in the prevalence of hypertension, type 2 diabetes (T2DM) and obesity, which are known risk factors for CVD is also observed in LMICs.[7 8]

Altered myocardial structure and function have received less attention as causes of cardiac death than ischaemic heart disease (IHD). Left ventricular hypertrophy (LVH) increases the future risk of heart failure and death[9] through volume overload, pressure overload and myocyte loss.[10 11] LVH is usually asymptomatic for several years before the development of congestive heart failure. Obesity, hypertension, T2DM and IHD initiate LV remodelling and dysfunction, and enhance progression to heart failure.[11 12] The prospective CARDIA study showed that higher blood pressure, BMI, waist circumference, cholesterol, triglyceride and glucose concentrations at age 18-30 years are risk factors for LV diastolic dysfunction 5-10 years later.[13] However, these traditional risk factors explain less than half the variability in echocardiographic parameters in population studies.[14 15]

South Asians develop CVD and T2DM, often in combination, a decade earlier than white Caucasians.[16 17] Several factors contribute to this premature disease: i) lifestyle changes (the adoption of less healthy diets and reductions in physical activity, consequences of rapid socio-economic transition), ii) a characteristic pattern of risk factors (low HDL-cholesterol, elevated lipoprotein(a) and high insulin resistance[18 19] and iii) a 'thin-fat' body composition (muscle-thin but centrally adipose),[19 20] the latter two being evident even at birth.[21 22]

South Asian newborns have a low mean birth weight (2.6-2.7 kg) compared with white Caucasian babies[21 23] and frequently show sub-optimal growth and weight gain

For peer review only - http://bmjopen.bmj.com/site/about/guidelines.xhtml

during infancy.[24] Low birth and infant weight are risk factors for CVD mortality in adult life and have also been linked to a high risk of hypertension and T2DM.[25 26] It has been suggested that these associations reflect 'developmental programming', permanent structural and functional deficits in key metabolic organs (eg. liver, pancreas and kidneys) and tissues (eg. muscle) resulting from impaired nutrition during fetal and early childhood development.[27] Studies in India have linked lower birth weight to higher blood pressure, serum lipids, and abnormal glucose tolerance in children and adults.[28-30] The risks associated with lower birth weight appear to be increased on subsequent exposure to a higher plane of nutrition. Upward crossing of BMI centiles in childhood and adolescence is associated with increases in several CVD risk factors such as higher adult cholesterol, triglyceride and pro-inflammatory marker concentrations, and with an increased risk of obesity, T2DM, hypertension and metabolic syndrome (Figure 1).[29 31]

Early life factors may also influence cardiac structure and function. Growth restricted fetuses and newborns have impaired LV function,[32-34] thought to be caused by hypoxia, raised placental resistance and altered fetal circulation.[35] Studies relating birth weight to adolescent or adult LV size, in a variety of populations, have shown a positive association[36] or a non-significant inverse association.[37 38] In the latter two studies, low weight in infancy was associated with higher adult LV mass and concentric LVH. Several studies have shown that higher childhood BMI is associated with higher LV mass in childhood and adult life, and an increased risk of adult LVH.[36 39 40]

There is little population-based data on the prevalence of echocardiographic abnormalities among South Asians. Indian migrants to the UK present with heart failure younger than white British men and women.[41] The echocardiographic imaging of healthy individuals in the UK LOLIPOP study showed that South Asians have poorer diastolic function, threefold higher prevalence of LVH, and a greater degree of concentric remodelling.[42] The UK SABRE study showed that the impact of hyperglycaemia on LV mass and function was more adverse in South Asians.[15] One previous Indian study has investigated LV mass in relation to birth size; it found low mean LV mass compared with white Caucasian populations, and an association of longer birth length with higher adult LV mass.[43 44]

For peer review only - http://bmjopen.bmj.com/site/about/guidelines.xhtml

Thus, several factors along the lifecourse are related to adult LV hypertrophy and dysfunction, including greater adiposity, longer duration of adiposity, higher blood pressure and lipids, impaired glucose tolerance, and early life growth patterns (Figure 2). The current IndEcho study was designed to investigate these relationships in two large population-based Indian birth cohorts.

Hypotheses

- The prevalence of LVH and LV dysfunction will be related to current cardio-metabolic risk factors and those measured 12-16 years ago in young adulthood, and to smoking and lower physical activity.
- LVH and LV dysfunction will be associated with lower birth weight, lower weight in infancy (the first two post-natal years) and faster BMI gain during childhood and adolescence.
- The associations of LVH and LV dysfunction with adult cardiometabolic risk factors will be stronger in men and women who had lower birth or infant weight.

METHODS AND ANALYSIS

Study Design

Multi-centre observational cohort study

Study population

The IndEcho study started in November 2016 and will continue until 2019. Participants will be recruited from two Indian birth cohorts: the New Delhi Birth Cohort (NDBC)[29 45] and Vellore Birth Cohort (VBC).[46] Both were established in 1969-1973, originally to study maternal health and pregnancy outcomes. The participants were measured at various time points through infancy, childhood, adolescence and early adulthood to assess growth, and were subsequently followed up at approximately 30 and 40 years of age to measure a range of CVD risk markers. Flow charts of the different stages of follow-up and various measurements are provided in

For peer review only

For peer review only - http://bmjopen.bmj.com/site/about/guidelines.xhtml

Figure 3. Currently, the participants are aged 43-50 years and new cases of glucose intolerance, T2DM and hypertension are emerging alongside rapid transitions in life-style and socio-economic status. For the IndEcho study, we aim to recruit approximately 3,000 individuals from both cohorts combined.

The New Delhi Birth Cohort (NDBC)

In 1969-72, 20,755 married women of reproductive age living in a 12 km^2 area of South Delhi were recruited (Figure 3). The cohort included 8,181 singleton live births from 9,169 pregnancies among these women. Gestational age was derived from last menstrual period (LMP) dates. The birth weight, length and head circumference of the babies were recorded within 72 hours of birth (n=7,119), and thereafter 3 monthly up to the age of 12 months (n=4,104) and 6 monthly until the age of 21 years (n=2,892). An average 23 sets of measurements were recorded for each individual from birth until 21 years. The first adult study took place in 1998-2002; 2,584 (32%) of the original cohort were re-traced, of whom 1,526 men and women (then aged 28-31 years) participated in a study of CVD risk factors.[29] Data on their socio-economic status (SES), attained education, family history of disease, tobacco and alcohol consumption, diet and physical activity were obtained using standardised questionnaires. Clinical measurements included anthropometry, blood pressure, an electrocardiogram (ECG), and analysis of plasma glucose (during an oral glucose tolerance test), insulin, lipids and pro-inflammatory markers. In 2008 (at age 34-39 years) the same measurements were repeated in 1,149 cohort members and additionally, carotid intima media thickness (CIMT) and brachial artery endothelial function were measured[47] and body composition was assessed using dual-energy absorptiometry (DXA) in a sub-set. For the IndEcho study, we aim to recruit as many as possible of the 1,526 participants from the 1998-2002 follow-up.

The Vellore Birth Cohort (VBC)

From 1969-1973, 20,626 women of reproductive age were recruited within defined areas of Vellore town and adjoining rural villages in Tamil Nadu, in South India[48] (Figure 3). The areas of Vellore town were selected to represent different socio-economic groups. The cohort comprised 10,691 singleton live babies born to the

For peer review only - http://bmjopen.bmj.com/site/about/guidelines.xhtml

20,626 women recruited. Weight and length were recorded at birth (n=10,676) and subsequently in infancy (in the first 3 months, n=5,753), childhood (6-8 years, n=5,541) and adolescence (10-15 years, n=2,672). Gestational age was determined from the mother's LMP dates. Depending on available funding, VBC members had up to 3 measurements in the first 3 months, up to 2 measurements between 6 and 8 years, and up to 5 measurements between 10 and 15 years. The first adult follow-up took place in 1998-2002. Cohort members for whom all birth measurements were available (n=4,092) were re-traced to participate in an adult follow-up during 1998-2002. Of these 2,572 were contactable, and 2,218 men and women, then aged 26-32 years, consented to participate in a the study. Cardiovascular risk factors were assessed using a similar protocol to that for NDBC.[49] Subsequently in 2013-14, 1,080 participants (50.1% urban), aged 41-45 years took part in a body composition study, in which the same anthropometric and biochemical parameters were recorded along with detailed body composition using dual energy X-ray absorptiometry (DXA). For the IndEcho study, we aim to recruit as many as possible of the 2,218 VBC members who participated in the 1998-2002 follow-up.

Measurements in the IndEcho study

Measurements will include anthropometry (height, weight, waist and hip circumferences, skinfold thickness), body composition by DXA (Vellore only) and bioelectrical impedance, hand grip strength, blood pressure, biochemical measurements (an oral glucose tolerance test, fasting plasma insulin and lipids, and urinary albumin-creatinine ratio, as a measure of microalbuminuria). Life-style factors (tobacco and alcohol consumption, diet, physical activity, occupation and socio-economic status (SES)) will be reassessed. Standard questionnaires for diet (food-frequency questionnaire),[50] SES (standard of living (SLI) index)[51] and the Global Physical Activity Questionnaire (GPAQ)[52] will be used. Details of the methods used for these measurements are given in Table 1.

Impaired glucose tolerance, impaired fasting glucose and T2DM will be diagnosed based on fasting glucose concentration and glucose concentration 120 minutes after a 75 g oral glucose load, using WHO criteria.[53] Hypertension will be defined as systolic pressure $\geq$140 mmHg or diastolic pressure $\geq$90 mmHg or on treatment for

For peer review only - http://bmjopen.bmj.com/site/about/guidelines.xhtml

hypertension.[54] We will use International Diabetes Federation (IDF) criteria for the following outcomes:[55] overweight and obesity: BMI $\geq$25 and $\geq$30 kg/m^2 respectively; central obesity: a waist circumference $\geq$90 cm in men and $\geq$80 cm in women; hyper-triglyceridaemia: plasma triglyceride concentration $\geq$1.7 mmol/l; and low HDL-cholesterol: <1.03 mmol/l in men and <1.29 mmol/l in women.

Echocardiography

Cardiac chamber dimensions and systolic and diastolic function will be assessed using transthoracic echocardiography. All measurements will be performed according to the American and European Societies for Echocardiography guidelines for chamber quantification[56, 57] and left ventricular diastolic function.[58] The same machine (Philips CX50 Compact Xtreme, Bothell, USA) and transducer (C5-1 Purewave curved array transducer) will be used in both centres and images will be analysed using the Freeland digitizer and software packages (Alpharetta, USA). M-mode and 2-D echocardiography in the parasternal long axis, mid-papillary short axis and apical 2- and 4-chamber views, will be used to measure relative wall thickness (RWT, an index of wall thickness relative to internal dimensions). LV systolic dysfunction will be assessed using measurements of fractional shortening, ejection fraction, and global longitudinal strain (GLS). Ejection fraction will be measured using Simpson's biplane method in apical 2 and 4 chamber views. GLS will be calculated offline using speckle-tracking technology from the acquired 2-D; 2-, 3- and 4-chamber views using the in-built QLAB software in the CX50 echo machine. LV diastolic dysfunction will be assessed using mitral valve inflow velocities, mitral annular tissue doppler velocities, left atrial volume index, and tricuspid regurgitation jet velocity as per the guidelines.[58]

Scans will be performed by experienced echo technicians, two per centre, after a joint 2-day protocol-specific training session. Following this, the technical quality of scans will be checked by the senior cardiologist locally in each centre, and a set of 10 scans will be exchanged between centres for an independent quality assessment. Scans will be read by experienced cardiologists in each centre (AR in New Delhi and VST and AGA in Vellore). A random 10% of all scans recorded in the study will be exchanged and read by the other centre to assess inter-observer variability.

For peer review only - http://bmjopen.bmj.com/site/about/guidelines.xhtml

Carotid intimal media thickness (CIMT) measurement

Measurements of CIMT will be made bilaterally using high-resolution B-mode ultrasound with the same machine (Philips CX50 Compact Xtreme) and a L12-3 Hz broad band linear array transducer according to American Society for Echocardiography guidelines.[59] Each common carotid artery will be imaged in three different projections (anterior, lateral and posterior) proximal to the bifurcation. The presence or absence of plaque, defined as a focal structure that encroaches upon the arterial lumen by at least 0.5 mm or is more than 50% of the surrounding intima-media thickness or has a thickness greater than or equal to 1.5 mm will also be recorded.

Electrocardiogram

All participants will undergo a 12-lead ECG recording. ECGs will be reviewed by a cardiologist, and if there are changes suggestive of ischaemia, a detailed evaluation will be done using Minnesota Codes.[60]

Body composition assessments

Body composition (total lean mass, fat mass and fat percent) will be assessed using bioelectrical impedance in both centres (Tanita BC-418 in NDBC and Bodystat-2500 in VBC). In VBC, a DXA examination will be additionally performed (Hologic Discovery) to obtain total and depot specific lean mass, fat mass and total fat percentage. DXA body composition analysis was assessed among NDBC participants in 2009[61] and will not be re-examined in IndEcho.

A copy of the full protocol can be obtained from the corresponding author.

Sample size and analysis

We will target the 3,744 cohort members (1,526 from NDBC and 2,218 from VBC)

For peer review only - http://bmjopen.bmj.com/site/about/guidelines.xhtml

who participated in follow-ups between 1998 and 2002. From initial tracing in early 2016, a total of 3,500 individuals were re-contacted, among whom we expect approximately 1,250 in Delhi and 1,750 in Vellore to participate (total 3,000) in the current protocol.

Exposures in early life will include weight and length at birth; weight, height and BMI and independent conditional weight and height estimates of growth during infancy, childhood and adolescence, as previously described.[48] Exposures in young adult life will include lifestyle characteristics (diet, physical activity, smoking and alcohol consumption, SES, urban/rural residence and history of rural-urban migration (Vellore only)); anthropometry; lean and fat mass measured by DXA and bio-impedance; metabolic risk markers (plasma glucose, insulin, and lipids) and categorical abnormalities as defined above.

We will check the distributions of all variables and perform appropriate transformations. We will explore relationships between LV wall thickness and mass with current body size, and make appropriate adjustments, including the conventional adjustment for body surface area and height.[56] We will create categorical variables to represent LVH and ventricular dysfunction. We will examine associations of early life and young adult exposures with outcomes, using linear and logistic regression as appropriate, with and without adjustment for potential confounders and covariates (including age, sex, history of migration, current body size and composition). We will explore interactions between early life and young adult exposures and between early life exposures and current measures, using product terms. We will explore the role of young adult lifestyle and CVD risk markers as mediators of associations between early life exposures and outcomes. We will assess risk of bias by comparing available data between cohort members studied and not studied. We will examine the data from men and women, from Delhi and Vellore, and (in Vellore) among rural and urban participants separately, and assess heterogeneity of associations and scope for pooling.

Using tests at 5% significance, and a total sample size of 3,000, we will have 80% power to detect an association of 0.05 SDs of a continuous outcome (eg LV mass) per SD change in a continuous predictor (eg birth weight or conditional weight gain in infancy). For the Vellore cohort alone (based on N=1,750) this figure is 0.07 SDs and

For peer review only

for the Delhi cohort alone (based on N=1,250) it is 0.08 SDs. These figures compare favorably with the association shown between infant weight and adult LV mass in the Hertfordshire cohort (0.17 SD per SD change in infant weight).[26] For a binary outcome such as LV diastolic dysfunction (Grade 1 & 2), assuming 10% prevalence and a test at 5% significance level, we will have 80% power to detect an association of 0.17 log odds per SD change in a continuous predictor, equivalent to an odds ratio of 1.19 (Vellore 1.25, Delhi 1.30).

DISCUSSION

To the best of our knowledge, IndEcho will be the first study among South Asians to provide data linking birth size and childhood growth, as well as prior measures of CVD risk markers and lifestyle factors such as physical activity and smoking in young adulthood, with cardiac structure and function and in middle age. It will provide an opportunity to study interactions between early life, young adult and concurrent risk factors in relation to myocardial outcomes.

The main strengths of the study are that it includes participants from two large Indian birth cohorts that have serial childhood growth data, measured prospectively by trained research staff, and risk factors for CVD (including lifestyle risk factors) assessed 16-20 years ago. Harmonised methods will be used in both cohorts. The participants represent different socioeconomic strata and rural/urban settings, making them representative of the general Indian population, and allowing assessment of the impact of economic transition on cardiac health in Indians. At this age we will detect sub-clinical cardiac disease in an apparently healthy population where obesity, hypertension and T2DM are escalating. In 1998-2002 at age 26-32 years, the prevalence of obesity (BMI $\geq$30 kg/m^2), hypertension (systolic $\geq$140 mmHg or diastolic $\geq$90 mmHg or on treatment for hypertension) and diabetes (WHO criteria) was 11%, 8% and 4% respectively in NDBC[29] and 2%, 3% and 3% in VBC.[31] Approximately 5 years later, at 34-39 years, the prevalence had more than doubled in NDBC (23%, 26% and 9%).[45] In the 2013-14 VBC follow-up, the equivalent data (16%, 20% and 17%) showed a more than 5-fold increase in prevalence in just over a decade (unpublished data).

For peer review only

Like all birth cohorts of this age, an important limitation is loss to follow-up, mainly due to deaths in childhood, and migration out of the original study area. However, current cohort members were similar in early life to the rest of the original cohorts.[29 48] In order to create bias, the relationship between the predictors (eg. birth weight, or young adult diabetes) and outcomes (eg. LV mass) would have to differ between those studied and not studied. Another limitation is that, because this is a field-based study, echocardiography will be used, rather than cardiac MRI, which is the current gold standard method for measuring myocardial structure

The findings of the IndEcho study will help plan effective strategies in early life and/or young adulthood to prevent adult CVD in India and migrant South Asian populations. We expect that it will identify periods during early life when nutritional interventions may prevent later cardiac disease. As the prospective follow-up continues in future, and as cohort members start to develop cardiac disease, we will also be able to define the value of LV measurements for predicting cardiovascular morbidity and mortality, which will be the first such data from India.

ETHICS AND DISSEMINATION

IndEcho does not involve any invasive or risky procedures for participants. Patient information sheets and consent forms are made available in English and local languages, describing the purpose of the study and the various procedures in lay terms. Participation is voluntary and participants provide written informed consent before enrollment. The study has been approved by the respective ethics committees of the participating institutions and is registered as ISRCTN13432279. Findings from the study will be disseminated through presentations at both international and national conferences and results generated will be published in international peer-reviewed journals.

Author affiliations

The study will involve investigators based in India (New Delhi and Vellore) and the UK. The participating institutions in New Delhi, India include Sunder Lal Jain Hospital (Santosh K Bhargava, Bhaskar Singh), Sitaram Bhartia Institute of Science and

For peer review only - http://bmjopen.bmj.com/site/about/guidelines.xhtml

Research Institute (HPS Sachdev, Sikha Sinha), Centre for Chronic Disease Control & Public Health Foundation of India (Prabhakaran Dorairaj), All-India Institute of Medical Sciences (Ambuj Roy, Nikhil Tandon, Lakshmy Ramakrishnan). The representatives of the VBC from the Christian Medical College, Vellore include from the Departments of Biostatistics (Antonisamy, Prasanna S Premkumar, Sneha FX Princy), Endocrinology (Nihal Jacob Thomas, Thomas Paul, Prrathepa Asaithambi, Kanhaiya Agrawal) Clinical Biochemistry (Finney S Geethanjali), and Cardiology (Anoop George Alexander and Viji Thomson Samuel). The two UK-based institutions include The Oxford Centre for Diabetes, Endocrinology and Metabolism, Oxford University, Oxford, UK (Fredrik Karpe) and MRC Lifecourse Epidemiology Unit, University of Southampton, UK (Senthil K Vasan, Clive Osmond and Caroline Fall).

Acknowledgements

We thank the members of the NBDC and VBC, and their families, for their participation in research over more than 4 decades. We acknowledge the IndEcho field teams in Delhi and Vellore, which include administrative staff, field workers and supervisors, research officers, research nurses, phlebotomists, DXA and echo technicians, data management teams and data entry operators.

ADDITIONAL INFORMATION

Authors' contributors

SKV[1], AR[2], VTS[3], BA[4], SKB[5], AAG[6], CO[7], FK[8], HS[9], LR[10], NT[11], NJT[12], TVP[13], DP[14] and CHD[15] conceived the study and wrote the study protocol. BS[16], FSG[17], KA[18], PSP[19], PA[20], SFXP[21] and SS[22] will contribute significantly to the acquisition of the data. CO[7], SKV[1], FK[8], DP[14], SKB[5], HS[9], AR[2], VTS[3], AAG[6], BA[4], PSP[19], SFXP[21] and CHDF[15] significantly contributed to the planning of analyses of the data. SKV[1] and CHDF[15] drafted the first version of the manuscript and all authors revised the manuscript critically for important intellectual content. All authors reviewed and approved the final manuscript and agree to be accountable for all aspects of the work

Funding

The original cohort studies were supported by the National Center for Health

For peer review only

For peer review only - http://bmjopen.bmj.com/site/about/guidelines.xhtml

Statistics, USA and the Indian Council of Medical Research. The two earlier follow-up studies in young adult life were supported by the British Heart Foundation. The IndEcho study is supported by British Heart Foundation Clinical Research Grant, No. CRM:0022324.

Competing interests: None declared.

Ethics approval

The study has been approved by the research ethics committees of Sunder Lal Jain Hospital, New Delhi (13th August 2015; SLJ/IEC/1); Sitaram Bhartia Institute of Science and Research, New Delhi (23rd October 2015; IEC/SBSR/2015/1); Centre for Chronic Disease Control, New Delhi (no.50/7/TF-CVD/15-NCD-II); All-India Institute of Medical Sciences, New Delhi (21st October 2015, IEC/NP-410/09.10.2015); Christian Medical College, Vellore (22nd July 2015; IRB 9548[OBSERV]) and the Faculty of Medicine, University of Southampton, UK (11th April 2016; RE 18694).

Provenance and peer review Not commissioned; externally peer reviewed.

Data sharing statement Data collection for this study is ongoing.

For peer review only

References

1. Mortality GBD, Causes of Death C. Global, regional, and national life expectancy,all cause mortality, and cause-specific mortality for 249 causes of death, 1980-2015: a systematic analysis for the Global Burden of Disease Study 2015. *Lancet* 2016;388(10053):1459-544.
2. Disease GBD, Injury I, Prevalence C. Global, regional, and national incidence, prevalence, and years lived with disability for 310 diseases and injuries, 1990-2015: a systematic analysis for the Global Burden of Disease Study 2015. *Lancet* 2016;388(10053):1545-602.
3. Roth GA, Johnson C, Abajobir A, et al. Global, Regional, and National Burden of Cardiovascular Diseases for 10 Causes, 1990 to 2015. *J Am Coll Cardiol* 2017;70(1):1-25.
4. McKeigue PM, Miller GJ, Marmot MG. Coronary heart disease in south Asians overseas: a review. *J Clin Epidemiol* 1989;42(7):597-609.
5. Wild S, McKeigue P. Cross sectional analysis of mortality by country of birth in England and Wales, 1970-92. *BMJ* 1997;314(7082):705-10.
6. Yusuf S, Reddy S, Ounpuu S, et al. Global burden of cardiovascular diseases: Part II: variations in cardiovascular disease by specific ethnic groups and geographic regions and prevention strategies. *Circulation* 2001;104(23):2855-64.
7. Lim SS, Vos T, Flaxman AD, et al. A comparative risk assessment of burden of disease and injury attributable to 67 risk factors and risk factor clusters in 21 regions, 1990-2010: a systematic analysis for the Global Burden of Disease Study 2010. *Lancet* 2012;380(9859):2224-60.
8. Collaboration NCDRF. Worldwide trends in diabetes since 1980: a pooled analysis of 751 population-based studies with 4.4 million participants. *Lancet* 2016;387(10027):1513-30.
9. Levy D, Garrison RJ, Savage DD, et al. Left ventricular mass and incidence of coronary heart disease in an elderly cohort. The Framingham Heart Study. *Ann Intern Med* 1989;110(2):101-7.
10. Abel ED, Litwin SE, Sweeney G. Cardiac remodeling in obesity. *Physiol Rev* 2008;88(2):389-419.
11. Boudina S, Abel ED. Diabetic cardiomyopathy revisited. *Circulation* 2007;115(25):3213-23.
12. Lauer MS, Anderson KM, Kannel WB, et al. The impact of obesity on left ventricular mass and geometry. The Framingham Heart Study. *JAMA* 1991;266(2):231-6.
13. Desai CS, Colangelo LA, Liu K, et al. Prevalence, prospective risk markers, and prognosis associated with the presence of left ventricular diastolic dysfunction in young adults: the coronary artery risk development in young adults study. *Am J Epidemiol* 2013;177(1):20-32.

For peer review only

14. Schirmer H, Lunde P, Rasmussen K. Mitral flow derived Doppler indices of left ventricular diastolic function in a general population; the Tromso study. *Eur Heart J* 2000;21(16):1376-86.
15. Tillin T, Hughes AD, Mayet J, et al. The relationship between metabolic risk factors and incident cardiovascular disease in Europeans, South Asians, and African Caribbeans: SABRE (Southall and Brent Revisited) -- a prospective population-based study. *J Am Coll Cardiol* 2013;61(17):1777-86.
16. Prabhakaran D, Jeemon P, Roy A. Cardiovascular Diseases in India: Current Epidemiology and Future Directions. *Circulation* 2016;133(16):1605-20.
17. Bellary S, O'Hare JP, Raymond NT, et al. Premature cardiovascular events and mortality in south Asians with type 2 diabetes in the United Kingdom Asian Diabetes Study - effect of ethnicity on risk. *Curr Med Res Opin* 2010;26(8):1873-9.
18. Bilen O, Kamal A, Virani SS. Lipoprotein abnormalities in South Asians and its association with cardiovascular disease: Current state and future directions. *World J Cardiol* 2016;8(3):247-57.
19. McKeigue PM, Shah B, Marmot MG. Relation of central obesity and insulin resistance with high diabetes prevalence and cardiovascular risk in South Asians. *Lancet* 1991;337(8738):382-6.
20. Raji A, Seely EW, Arky RA, et al. Body fat distribution and insulin resistance in healthy Asian Indians and Caucasians. *J Clin Endocrinol Metab* 2001;86(11):5366-71.
21. Yajnik CS, Fall CH, Coyaji KJ, et al. Neonatal anthropometry: the thin-fat Indian baby. The Pune Maternal Nutrition Study. *Int J Obes Relat Metab Disord* 2003;27(2):173-80.
22. Yajnik CS, Lubree HG, Rege SS, et al. Adiposity and hyperinsulinemia in Indians are present at birth. *J Clin Endocrinol Metab* 2002;87(12):5575-80.
23. Seaton SE, Yadav KD, Field DJ, et al. Birthweight centile charts for South Asian infants born in the UK. *Neonatology* 2011;100(4):398-403.
24. Joglekar CV, Fall CH, Deshpande VU, et al. Newborn size, infant and childhood growth, and body composition and cardiovascular disease risk factors at the age of 6 years: the Pune Maternal Nutrition Study. *Int J Obes (Lond)* 2007;31(10):1534-44.
25. Curhan GC, Willett WC, Rimm EB, et al. Birth weight and adult hypertension, diabetes mellitus, and obesity in US men. *Circulation* 1996;94(12):3246-50.
26. Osmond C, Barker DJ, Winter PD, et al. Early growth and death from cardiovascular disease in women. *BMJ* 1993;307(6918):1519-24.
27. Barker DJ. Fetal programming of coronary heart disease. *Trends Endocrinol Metab* 2002;13(9):364-8.
28. Stein CE, Fall CH, Kumaran K, et al. Fetal growth and coronary heart disease in south India. *Lancet* 1996;348(9037):1269-73.
29. Bhargava SK, Sachdev HS, Fall CH, et al. Relation of serial changes in childhood body-mass index to impaired glucose tolerance in young adulthood. *N Engl J Med* 2004;350(9):865-75.

For peer review only

30. Bavdekar A, Yajnik CS, Fall CH, et al. Insulin resistance syndrome in 8-year-old Indian children: small at birth, big at 8 years, or both? *Diabetes* 1999;48(12):2422-9.
31. Raghupathy P, Antonisamy B, Geethanjali FS, et al. Glucose tolerance, insulin resistance and insulin secretion in young south Indian adults: Relationships to parental size, neonatal size and childhood body mass index. *Diabetes Res Clin Pract* 2010;87(2):283-92.
32. Crispi F, Bijnens B, Figueras F, et al. Fetal growth restriction results in remodeled and less efficient hearts in children. *Circulation* 2010;121(22):2427-36.
33. Crispi F, Figueras F, Cruz-Lemini M, et al. Cardiovascular programming in children born small for gestational age and relationship with prenatal signs of severity. *Am J Obstet Gynecol* 2012;207(2):121 e1-9.
34. Tsyvian P, Malkin K, Artemieva O, et al. Cardiac ventricular performance in the appropriate- for-gestational age and small-for-gestational age fetus: relation to regional cardiac non-uniformity and peripheral resistance. *Ultrasound Obstet Gynecol* 2002;20(1):35-41.
35. Thornburg KL, Louey S. Fetal roots of cardiac disease. *Heart* 2005;91(7):867-8.
36. Hietalampi H, Pahkala K, Jokinen E, et al. Left ventricular mass and geometry in adolescence: early childhood determinants. *Hypertension* 2012;60(5):1266-72.
37. Vijayakumar M, Fall CH, Osmond C, et al. Birth weight, weight at one year, and left ventricular mass in adult life. *Br Heart J* 1995;73(4):363-7.
38. Zureik M, Bonithon-Kopp C, Lecomte E, et al. Weights at birth and in early infancy, systolic pressure, and left ventricular structure in subjects aged 8 to 24 years. *Hypertension* 1996;27(3 Pt 1):339-45.
39. Li X, Li S, Ulusoy E, et al. Childhood adiposity as a predictor of cardiac mass in adulthood: the Bogalusa Heart Study. *Circulation* 2004;110(22):3488-92.
40. Sivanandam S, Sinaiko AR, Jacobs DR, Jr., et al. Relation of increase in adiposity to increase in left ventricular mass from childhood to young adulthood. *Am J Cardiol* 2006;98(3):411-5.
41. Blackledge HM, Newton J, Squire IB. Prognosis for South Asian and white patients newly admitted to hospital with heart failure in the United Kingdom: historical cohort study. *BMJ* 2003;327(7414):526-31.
42. Chahal NS, Lim TK, Jain P, et al. Ethnicity-related differences in left ventricular function, structure and geometry: a population study of UK Indian Asian and European white subjects. *Heart* 2010;96(6):466-71.
43. Kumaran K, Fall CH, Martyn CN, et al. Left ventricular mass and arterial compliance: relation to coronary heart disease and its risk factors in South Indian adults. *Int J Cardiol* 2002;83(1):1-9.
44. Kumaran K, Fall CH, Martyn CN, et al. Blood pressure, arterial compliance, and left ventricular mass: no relation to small size at birth in south Indian adults. *Heart* 2000;83(3):272-7.

For peer review only

For peer review only - http://bmjopen.bmj.com/site/about/guidelines.xhtml

45. Huffman MD, Prabhakaran D, Osmond C, et al. Incidence of cardiovascular risk factors in an Indian urban cohort results from the New Delhi birth cohort. *J Am Coll Cardiol* 2011;57(17):1765-74.
46. Antonisamy B, Raghupathy P, Christopher S, et al. Cohort Profile: the 1969-73 Vellore birth cohort study in South India. *Int J Epidemiol* 2009;38(3):663-9.
47. Khalil A, Huffman MD, Prabhakaran D, et al. Predictors of carotid intima-media thickness and carotid plaque in young Indian adults: the New Delhi birth cohort. *Int J Cardiol* 2013;167(4):1322-8.
48. Antonisamy B, Vasan SK, Geethanjali FS, et al. Weight Gain and Height Growth during Infancy, Childhood, and Adolescence as Predictors of Adult Cardiovascular Risk. *J Pediatr* 2017;180:53-61 e3.
49. Raghupathy P, Antonisamy B, Fall CH, et al. High prevalence of glucose intolerance even among young adults in south India. *Diabetes Res Clin Pract* 2007;77(2):269-79.
50. National Institute of Nutrition (NIN). National Nutrition Monitoring Bureau Report of Repeat Surveys (1998-90). In: Indian Council of Medical Research. Hyderabad I, ed., 1991.
51. IIPS and Macro International (2000) National Family Health Survey (NFHS-2). West Bengal: International Institute of Population Sciences, Mumbai., 1998-99.
52. Bull FC, Maslin TS, Armstrong T. Global physical activity questionnaire (GPAQ): nine country reliability and validity study. *J Phys Act Health* 2009;6(6):790-804.
53. World Health Organization: Definition, Diagnosis and Classification of Diabetes Mellitus and its. Complications: Report of a WHO Consultation. 1999.
54. James PA, Oparil S, Carter BL, et al. 2014 evidence-based guideline for the management of high blood pressure in adults: report from the panel members appointed to the Eighth Joint National Committee (JNC 8). *JAMA* 2014;311(5):507-20.
55. Alberti KGMM, Zimmet P, Shaw J. The metabolic syndrome - a new worldwide definition. *Lance*t 2005;366(9491):1059-62.
56. Lang RM, Badano LP, Mor-Avi V, et al. Recommendations for cardiac chamber quantification by echocardiography in adults: an update from the American Society of Echocardiography and the European Association of Cardiovascular Imaging. *J Am Soc Echocardiogr* 2015;28(1):1-39 e14.
57. Lang RM, Bierig M, Devereux RB, et al. Recommendations for chamber quantification. *Eur J Echocardiogr* 2006;7(2):79-108.
58. Nagueh SF, Smiseth OA, Appleton CP, et al. Recommendations for the Evaluation of Left Ventricular Diastolic Function by Echocardiography: An Update from the American Society of Echocardiography and the European Association of Cardiovascular Imaging. *J Am Soc Echocardiogr* 2016;29(4):277-314.
59. Stein JH, Korcarz CE, Hurst RT, et al. Use of carotid ultrasound to identify subclinical vascular disease and evaluate cardiovascular disease risk: a

For peer review only - http://bmjopen.bmj.com/site/about/guidelines.xhtml

consensus statement from the American Society of Echocardiography Carotid Intima-Media Thickness Task Force. Endorsed by the Society for Vascular Medicine. *J Am Soc Echocardiogr* 2008;21(2):93-111; quiz 89-90.

60. Prineas RJ CR, Blackburn H. The Minnesota code manual of electrocardiographic findings: standards and procedures for measurement and classification. Boston, Wright 1982.

61. Tandon N, Fall CH, Osmond C, et al. Growth from birth to adulthood and peak bone mass and density data from the New Delhi Birth Cohort. *Osteoporos Int* 2012;23(10):2447-59.

For peer review only

Table 1 IndEcho study procedures and platforms used

Study-related procedures	Methods /platforms used
Questionnaire assessments	
Diet	Food Frequency Questionnaire (FFQ)
Physical activity	General Physical Activity Questionnaire (GPAQ)
Socioeconomic status	NFHS Standard of Living Index
Smoking	NFHS-2 Household Questionnaire
Alcohol consumption	NFHS-2 Household Questionnaire
Anthropometry	
Height	Stadiometer
Weight	Digital weighing scales
Waist circumference	Non-stretchable tape
Hip circumference	Non-stretchable tape
Blood pressure	Omron M3
Biochemistry	
Glucose - fasting	Enzymatic method (autoanalyser)
Glucose – 120 min	Enzymatic method (autoanalyser)
Insulin - fasting	Vellore: Radio-fluorimetric method<br>Delhi: Chemiluminescence immunoassay
Cholesterol	CHOD-PAP Enzymatic colorimetric method
Triglycerides	GPO-PAP Enzymatic colorimetric method
HDL-cholesterol	Direct – two step enzymatic
LDL-cholesterol	Direct – Enzymatic colorimetric method
Urinary ACR	Jaffe Method
Skin fold thickness	John Bull/Harpenden skinfold calliper
Bioimpedence	Tanita BC-418/ Bodystat 2500
Hand Grip	JAMAR dynamometer
DXA*	Hologic Discovery
Electrocardiogram (ECG)	
Echocardiography	Philips CX50 Compact Xtreme system
CIMT#	Philips CX50 Compact Xtreme system

FFQ developed by the National Institute of Nutrition, Hyderabad[51]
SES Questionnaire developed by the National Family Health Survey (NFHS-2), 1998-99[52]
GPAQ: Global Physical Activity Questionnaire developed by WHO[53]
*DXA- Dual Energy X-ray absorptiometry
#CIMT- Carotid Intimal Media thickness

For peer review only

Figure 1 BMI SD scores from birth to adulthood for participants in each cohort who developed impaired glucose tolerance (IGT) or diabetes (N=219/1562 in NDBC and 424/2218 in VBC) in adult life relative to the whole cohort (dashed zero line)

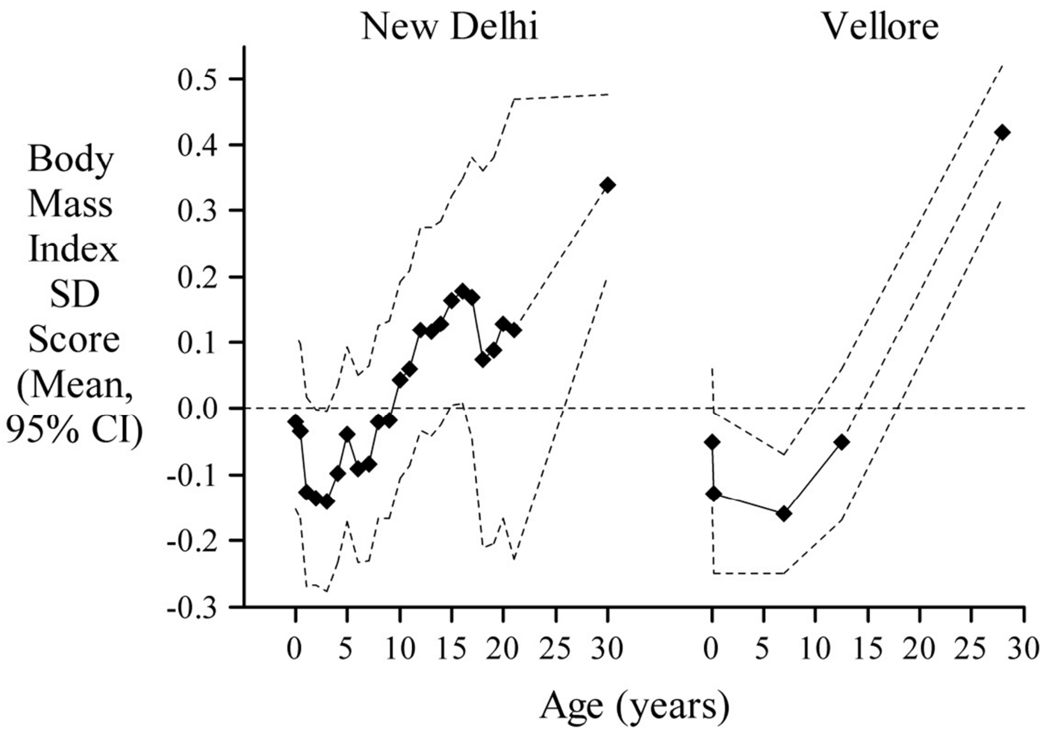

For peer review only - http://bmjopen.bmj.com/site/about/guidelines.xhtml

Figure 2: Pathways to altered left ventricular structure and function that will be investigated in IndEcho

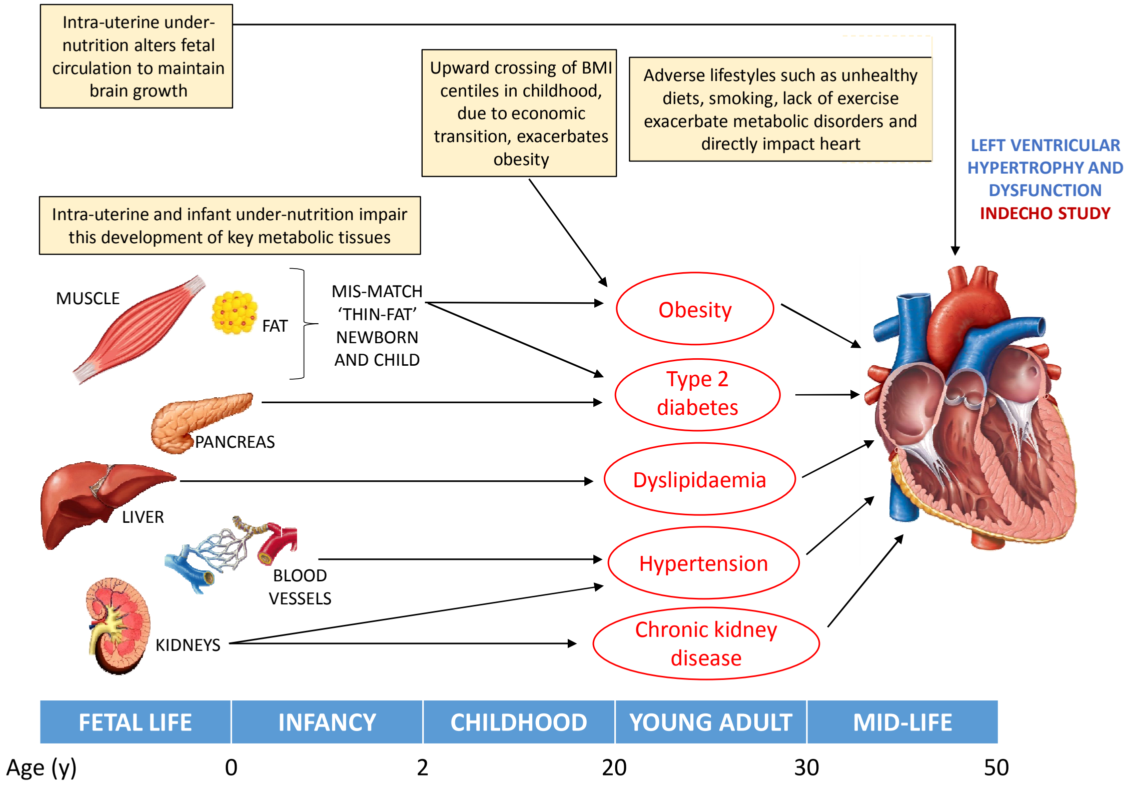

Factors contributing to left ventricular hypertrophy. Intra-uterine under-nutrition alters the fetal circulation, which may have direct and persistent effects on ventricular structure. Intra-uterine and infant under-nutrition impairs the development of key metabolic tissues (muscle, pancreas, liver, blood vessels and kidneys) during critical periods of growth, and increases adipose tissue deposition, leading to the 'thin-fat' phenotype, and later obesity, T2DM, dyslipidaemia, hypertension and chronic renal disease, which adversely impact on LV size and function

Figure 3 The New Delhi Birth Cohort and Vellore Birth Cohort

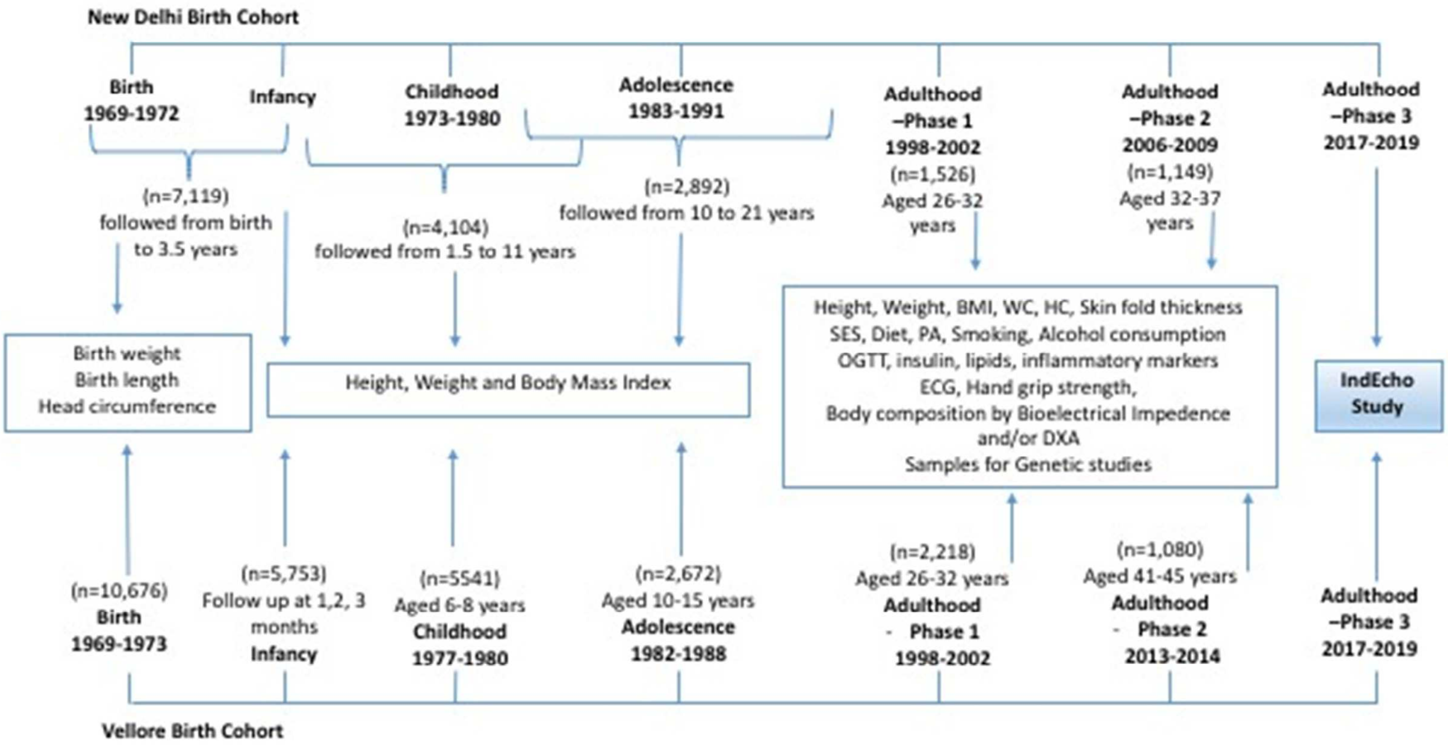

3

BMJ Open

The IndEcho study: Cohort study investigating birth size, childhood growth and young adult cardiovascular risk factors as predictors of mid-life myocardial structure and function in South Asians

Journal:	*BMJ Open*
Manuscript ID	bmjopen-2017-019675.R1
Article Type:	Protocol
Date Submitted by the Author:	19-Jan-2018
Complete List of Authors:	Vasan, Senthil; University of Oxford, Radcliffe Department of Medicine; University of Southampton, MRC Lifecourse Epidemiology Unit<br>Roy, Ambuj; All India Institute of Medical Sciences, Cardiology<br>Samuel, Viji; Christian Medical College and Hospital Vellore, Cardiology<br>Antonisamy, Belavendra; Christian Medical College and Hospital Vellore, Biostatistics<br>Bhargava, Santosh; Sunder Lal Jain Hospital<br>Alex, Anoop; Christian Medical College and Hospital Vellore, Cardiology<br>Singh, Bhaskar; Sunder Lal Jain Hospital<br>Osmond, Clive; University of Southampton , MRC Lifecourse Epidemiology Unit<br>Geethanjali, Finney; Christian Medical College and Hospital Vellore, Clinical Biochemistry<br>Karpe, Fredrik; University of Oxford , Oxford center for Diabetes, Endocrinology and Metabolism<br>Sachdev, Harshpal; Sitaram Bhartia Institute of Science and Research, Department of Paediatrics<br>Agrawal, Kanhaiya ; Christian Medical College and Hospital Vellore, Endocrinology<br>Ramakrishnan, Lakshmy; All India Institute of Medical Sciences,<br>Tandon, Nikhil; All India Institute of Medical Sciences,<br>THOMAS, NIHAL; CHRISTIAN MEDICAL COLLEGE, ENDOCRINOLOGY<br>Premkumar, Prasanna; Christian Medical College and Hospital Vellore, Biostatistics<br>Asaithambi, Prrathepa ; Christian Medical College and Hospital Vellore, Endocrinology<br>Princy , Sneha; Christian Medical College and Hospital Vellore, Biostatistics<br>Sinha , Sikha; Sitaram Bhartia Institute of Science and Research<br>PAUL, THOMAS; CHRISTIAN MEDICAL COLLEGE, ENDOCRINOLOGY&DIABETES<br>Prabhakaran, Dorairaj; Centre for Chronic Disease Control, ; Public Health Foundation of India,<br>Fall, Caroline ; University of Southampton, MRC Lifecourse Epidemiology Unit
**Primary Subject Heading**:	Cardiovascular medicine
Secondary Subject Heading:	Diabetes and endocrinology, Epidemiology, Global health, Cardiovascular

	medicine
Keywords:	IndEcho, South Asians, Echocardiography < CARDIOLOGY, Adiposity, Left ventricular mass and function, Birth weight and early growth

SCHOLARONE™ Manuscripts

For peer review only

The IndEcho study: Cohort study investigating birth size, childhood growth and young adult cardiovascular risk factors as predictors of mid-life myocardial structure and function in South Asians

Senthil K Vasan[1,2], Ambuj Roy[3,4], Viji Thomson Samuel[5], Belavendra Antonisamy[5], Santosh K Bhargava[6], Anoop George Alex[5], Bhaskar Singh[6], Clive Osmond[1], Finney S Geethanjali[5], Fredrik Karpe[2], Harshpal Sachdev[7], Kanhaiya Agrawal[5], Lakshmy Ramakrishnan[4], Nikhil Tandon[4], Nihal Jacob Thomas[5], Prasanna S Premkumar[5], Prrathepa Asaithambi[5], Sneha F.X. Princy[5], Sikha Sinha[7], Thomas Vizhalil Paul[5], Dorairaj Prabhakaran[3,8], Caroline HD Fall[1]

[1]MRC Lifecourse Epidemiology Unit, University of Southampton, Southampton General Hospital, Southampton UK
[2]Oxford center for Diabetes, Endocrinology and Metabolism, Radcliffe Department of Medicine, University of Oxford, Oxford, UK
[3]Centre for Chronic Disease Control, Gurgaon, New Delhi, India
[4]All-India Institute of Medical Sciences, New Delhi, India
[5]Christian Medical College, Vellore, India
[6]Sunder Lal Jain Hospital, New Delhi, India
[7]Sitaram Bhartia Institute of Science and Research, New Delhi, India
[8]Public Health Foundation of India, Gurgaon, India

Address for correspondence:

Caroline HD Fall
MRC Lifecourse Epidemiology Unit
University of Southampton
Southampton General Hospital
Tremona Road, Southampton, Hampshire, UK
SO16 6YD
Tel: +44 2380 777624
Fax: +44 2380 704021
Email: chdf@mrc.soton.ac.uk

Word count: Abstract. 299/300; Text 3746/4000 words; Tables 2; Figures 3

Key words: IndEcho, South Asians, Echocardiography, Left ventricular mass, Left ventricular function, Adiposity, Cardiovascular disease risk markers, Birth weight, Child growth

For peer review only

ABSTRACT

Introduction South Asians have high rates of cardiovascular disease (CVD) and its risk factors (hypertension, diabetes, dyslipidaemia and central obesity). Left ventricular (LV) hypertrophy and dysfunction are features of these disorders and important predictors of CVD mortality. Lower birth and infant weight, and greater childhood weight gain are associated with increased adult CVD mortality but there are few data on their relationship to LV function. The IndEcho study will examine associations of birth size, growth during infancy, childhood and adolescence, and CVD risk factors in young adulthood with mid-life cardiac structure and function in South Asian Indians.

Methods and analysis We propose to study approximately 3,000 men and women aged 43-50 years from two birth cohorts established in 1969-1973: the New Delhi Birth Cohort (NDBC; n=1,508) and Vellore Birth Cohort (VBC; n=2,156). They had serial measurements of weight and height from birth to early adulthood. CVD risk markers (body composition, blood pressure, glucose tolerance and lipids) and lifestyle characteristics (tobacco and alcohol consumption, physical activity, socio-economic status) were assessed at age ~30 years. Clinical measurements in IndEcho will include anthropometry, blood pressure, biochemistry (glucose, fasting insulin and lipids, urinary albumin/creatinine ratio) and body composition by dual energy X-ray absorptiometry and bioelectrical impedance. Outcomes are LV mass and indices of LV systolic and diastolic function assessed by 2-D and Doppler echocardiography, carotid intima media thickness (cIMT) and electrocardiographic indicators of ischaemia. Regression and conditional growth models, adjusted for potential confounders, will be used to study associations of childhood and young adult exposures with these cardiovascular outcomes.

Ethics and dissemination The study has been approved by the Health Ministry Steering Committee, Government of India, and institutional ethics committees of participating centres in India, and the University of Southampton, UK. Results will be disseminated through scientific meetings and peer-reviewed journals.

Registration ISRCTN13432279.

For peer review only

Strengths and limitations of the study

- The strengths include the longitudinal study design with early growth data, which allows examination of the relationship of size at birth, childhood growth and cardiovascular disease risk markers in young adult life with myocardial structure and function in mid-life in South Asians.
- The two cohorts represent both rural and urban populations, north and south India, and diverse socio-economic strata.
- The study will use harmonised methods to measure anthropometry, biochemical risk factors and to characterise lifestyle factors.
- A limitation of the study is attrition of the cohorts due to deaths and migration (mainly in childhood).
- Because this is a field-based study, echocardiography will be used, rather than cardiac MRI, which is the current gold standard method for measuring myocardial structure.

For peer review only

INTRODUCTION

Background and Rationale

The emerging epidemic of cardiovascular disease (CVD) in transitioning populations means that low and middle-income countries (LMICs) contribute a larger proportion to the global burden of CVD (~8-9 million deaths per year) than high income countries (~5 million).[1-3] Migrants from LMICs to high income countries experience an excess of CVD compared with indigenous populations.[4-6] A parallel increase in the prevalence of hypertension, type 2 diabetes (T2DM) and obesity, which are known risk factors for CVD is also observed in LMICs.[7 8]

Altered myocardial structure and function have received less attention as causes of cardiac death than ischaemic heart disease (IHD). Left ventricular hypertrophy (LVH) increases the future risk of heart failure and death[9] through volume overload, pressure overload and myocyte loss.[10 11] LVH is usually asymptomatic for several years before the development of congestive heart failure. Obesity, hypertension, T2DM and IHD initiate LV remodelling and dysfunction, and enhance progression to heart failure.[11 12] The prospective CARDIA study showed that higher blood pressure, BMI, waist circumference, cholesterol, triglyceride and glucose concentrations at age 18-30 years are risk factors for LV diastolic dysfunction 5-10 years later.[13] However, these traditional risk factors explain less than half the variability in echocardiographic parameters in population studies.[14 15]

South Asians develop CVD and T2DM, often in combination, a decade earlier than white Caucasians.[16 17] Several factors contribute to this premature disease: i) lifestyle changes (the adoption of less healthy diets and reductions in physical activity, consequences of rapid socio-economic transition), ii) a characteristic pattern of risk factors (low HDL-cholesterol, elevated lipoprotein(a) and high insulin resistance[18 19] and iii) a ‘thin-fat’ body composition (low muscle mass but centrally adipose),[19 20] the latter two being evident even at birth.[21 22]

South Asian newborns have a low mean birth weight (2.6-2.7 kg) compared with white Caucasian babies[21 23] and frequently show sub-optimal growth and weight gain

For peer review only - http://bmjopen.bmj.com/site/about/guidelines.xhtml

during infancy.[24] Low birth and infant weight are risk factors for CVD mortality in adult life and have also been linked to a high risk of hypertension and T2DM.[25 26] It has been suggested that these associations reflect ‘developmental programming’, permanent structural and functional deficits in key metabolic organs (eg. liver, pancreas and kidneys) and tissues (eg. muscle) resulting from impaired nutrition during fetal and early childhood development.[27] Studies in India have linked lower birth weight to higher blood pressure, serum lipids, and abnormal glucose tolerance in children and adults.[28-30] The risks associated with lower birth weight appear to be increased on subsequent exposure to greater child or adult weight gain. Upward crossing of BMI centiles in childhood and adolescence is associated with increases in several CVD risk factors such as higher adult cholesterol, triglyceride and pro-inflammatory marker concentrations, and with an increased risk of obesity, impaired glucose tolerance or T2DM (Figure 1), hypertension and metabolic syndrome.[29 31]

Early life factors may also influence cardiac structure and function. Growth restricted fetuses and newborns have impaired LV function,[32-34] thought to be caused by hypoxia, raised placental resistance and altered fetal circulation.[35] Studies relating birth weight to adolescent or adult LV size, in a variety of populations, have shown a positive association[36] or a non-significant inverse association.[37 38] In the latter two studies, low weight in infancy was associated with higher adult LV mass and concentric LVH. Several studies have shown that higher childhood BMI is associated with higher LV mass in childhood and adult life, and an increased risk of adult LVH.[36 39 40]

There are few population-based data on the prevalence of echocardiographic abnormalities among South Asians. Indian migrants to the UK present with heart failure younger than white British men and women.[41] The echocardiographic imaging of healthy individuals in the UK LOLIPOP study showed that South Asians have poorer diastolic function, threefold higher prevalence of LVH, and a greater degree of concentric remodelling.[42] The UK SABRE study showed that the impact of hyperglycaemia on LV mass and function was more adverse in South Asians.[15] One previous Indian study has investigated LV mass in relation to birth size; it found low mean LV mass compared with white Caucasian populations, and an association of longer birth length with higher adult LV mass.[43 44]

For peer review only - http://bmjopen.bmj.com/site/about/guidelines.xhtml

Thus, several factors along the lifecourse are related to adult LV hypertrophy and dysfunction, including greater adiposity, longer duration of adiposity, higher blood pressure and lipids, impaired glucose tolerance, and early life growth patterns (Figure 2). The current IndEcho study was designed to investigate these relationships in two large population-based Indian birth cohorts.

Hypotheses

- The prevalence of LVH and LV dysfunction, and cIMT, will be positively related to current mid-life cardio-metabolic risk factors

- After adjusting for current cardio-metabolic risk factors, LVH, LV dysfunction and cIMT will be positively related to cardio-metabolic risk factors measured 12-16 years ago in young adulthood.

- LVH, LV dysfunction and higher cIMT will be associated with lower birth weight, lower weight in infancy (the first two post-natal years) and faster BMI gain during childhood and adolescence.

- The associations of LVH, LV dysfunction and cIMT with adult cardiometabolic risk factors will be stronger in men and women who had lower birth or infant weight.

METHODS AND ANALYSIS

Study Design

Multi-centre observational cohort study

Study population

The IndEcho study started in November 2016 and will continue until 2019. Participants will be recruited from two Indian birth cohorts: the New Delhi Birth Cohort (NDBC)[29 45] and Vellore Birth Cohort (VBC).[46] Both were established in 1969-1973, originally to study maternal health and pregnancy outcomes. The participants were measured at various time points through infancy, childhood, adolescence and

For peer review only - http://bmjopen.bmj.com/site/about/guidelines.xhtml

early adulthood to assess growth, and were subsequently followed up at approximately 30 and 40 years of age to measure a range of CVD risk markers. Flow charts of the different stages of follow-up and various measurements are provided in Figure 3. Currently, the participants are aged 43-50 years and new cases of glucose intolerance, T2DM and hypertension are emerging alongside rapid transitions in life-style and socio-economic status. For the IndEcho study, we aim to recruit approximately 3,000 individuals from both cohorts combined.

The New Delhi Birth Cohort (NDBC)

In 1969-72, 20,755 married women of reproductive age living in a 12 km^2 area of South Delhi were recruited (Figure 3). The cohort included 8,181 singleton live births from 9,169 pregnancies among these women. Gestational age was derived from last menstrual period (LMP) dates. The birth weight, length and head circumference of the babies were recorded within 72 hours of birth (n=7,119), and thereafter 3 monthly up to the age of 12 months (n=4,104) and 6 monthly until the age of 21 years (n=2,892). An average 23 sets of measurements were recorded for each individual from birth until 21 years. The first adult study took place in 1998-2002; 2,584 (32%) of the original cohort were re-traced, of whom 1,526 men and women (then aged 28-31 years) participated in a study of CVD risk factors.[29] Data on their socio-economic status (SES), attained education, family history of disease, tobacco and alcohol consumption, diet and physical activity were obtained using standardised questionnaires. Clinical measurements included anthropometry, blood pressure, an electrocardiogram (ECG), and analysis of plasma glucose (during an oral glucose tolerance test), insulin, lipids and pro-inflammatory markers. In 2008 (at age 34-39 years) the same measurements were repeated in 1,100 cohort members and additionally, carotid intima media thickness (cIMT) and brachial artery endothelial function were measured[47] and body composition was assessed using dual-energy absorptiometry (DXA) in a sub-set. Excluding 18 known deaths (from a total of 1,526 who were followed up during 1998-2002), we aim to recruit as many as possible of the 1,508 alive NDBC participants.

The Vellore Birth Cohort (VBC)

For peer review only - http://bmjopen.bmj.com/site/about/guidelines.xhtml

From 1969-1973, 20,626 women of reproductive age were recruited within defined areas of Vellore town and adjoining rural villages in Tamil Nadu, in South India[48] (Figure 3). The areas of Vellore town were selected to represent different socio-economic groups. The cohort comprised 10,691 singleton live babies born to the 20,626 women recruited. Weight and length were recorded at birth (n=10,676) and subsequently in infancy (in the first 3 months, n=5,753), childhood (6-8 years, n=5,541) and adolescence (10-15 years, n=2,672). Gestational age was determined from the mother's LMP dates. Depending on available funding, VBC members had up to 3 measurements in the first 3 months, up to 2 measurements between 6 and 8 years, and up to 5 measurements between 10 and 15 years. The first adult follow-up took place in 1998-2002. Cohort members for whom all birth measurements were available (n=4,092) were re-traced to participate in an adult follow-up during 1998-2002. Of these 2,572 were contactable, and 2,218 men and women, then aged 26-32 years, consented to participate in the study. Cardiovascular risk factors were assessed using a similar protocol to that for NDBC.[49] Subsequently in 2013-14, 1,080 participants (50.1% urban), aged 41-45 years took part in a body composition study, in which the same anthropometric and biochemical parameters were recorded along with detailed body composition using dual energy X-ray absorptiometry (DXA). Excluding 62 known deaths (from a total of 2,218 who were followed up during 1998-2002), we aim to recruit as many as possible of the 2,156 alive VBC members.

The total number of participants in Phases 1 and 2 of adult follow-up in the NDBC and VBC, and reasons for losses to follow-up are summarized in Supplementary table 1. We will minimize loss to follow-up through the following algorithm, until all non-responders are accounted for: telephone calls/postal letters, meetings with neighbours by field workers, and contact with local municipality/postal services to track addresses of migrated individuals.

Measurements in the IndEcho study

Measurements will include anthropometry (height, weight, waist and hip circumferences, skinfold thickness), body composition by DXA (Vellore only) and bioelectrical impedance, hand grip strength, blood pressure, biochemical measurements (an oral glucose tolerance test, fasting plasma insulin and lipids, and

For peer review only - http://bmjopen.bmj.com/site/about/guidelines.xhtml

urinary albumin-creatinine ratio, as a measure of microalbuminuria). Details of the methods used for these measurements are given in Table 1. Life-style factors (tobacco and alcohol consumption, diet, physical activity, occupation and socio-economic status (SES)) will be reassessed. Standard questionnaires for diet (food-frequency questionnaire),[50] SES (standard of living (SLI) index),[51-53] Global Physical Activity Questionnaire (GPAQ),[54 55] smoking and alcohol consumption[56] will be used. Details on the key co-variates relating to life style are summarized in Supplementary table 2.

Impaired glucose tolerance, impaired fasting glucose and T2DM will be diagnosed based on fasting glucose concentration and glucose concentration 120 minutes after a 75 g oral glucose load, using WHO criteria.[57] Hypertension will be defined as systolic pressure $\geq$140 mmHg or diastolic pressure $\geq$90 mmHg or on treatment for hypertension.[58] We will use International Diabetes Federation (IDF) criteria for the following outcomes:[59] overweight and obesity: BMI $\geq$25 and $\geq$30 kg/m^2 respectively; central obesity: a waist circumference $\geq$90 cm in men and $\geq$80 cm in women; hyper-triglyceridaemia: plasma triglyceride concentration $\geq$1.7 mmol/l; and low HDL-cholesterol: <1.03 mmol/l in men and <1.29 mmol/l in women.

Echocardiography

Cardiac chamber dimensions and systolic and diastolic function will be assessed using transthoracic echocardiography. All measurements will be performed according to the American and European Societies for Echocardiography guidelines for chamber quantification[60 61] and left ventricular diastolic function.[62] The same machine (Philips CX50 Compact Xtreme, Bothell, USA) and transducer (C5-1 Purewave curved array transducer) will be used in both centres and images will be analysed using the Freeland digitizer and software packages (Alpharetta, USA). M-mode and 2-D echocardiography in the parasternal long axis, mid-papillary short axis and apical 2- and 4-chamber views, will be used to measure relative wall thickness (RWT, an index of wall thickness relative to internal dimensions). LVH will be defined based on any one of the following criteria: (i) LV mass >150 g in women and >200 g in men, (ii) posterior wall thickness >1.1 cm or (iii) LV mass indexed to BSA >95 g/m^2 in women and 115 g/m^2 in men.[61] Concentric LVH, which is an independent CVD risk

For peer review only - http://bmjopen.bmj.com/site/about/guidelines.xhtml

factor, particularly in hypertensive patients, will be defined as increased relative wall thickness (RWT) >0.42 in the presence of normal LV mass. LV systolic dysfunction will be assessed using measurements of fractional shortening, ejection fraction, and global longitudinal strain (GLS). Ejection fraction will be measured using Simpson's biplane method in apical 2 and 4 chamber views. GLS will be calculated offline using speckle-tracking technology from the acquired 2-D; 2-, 3- and 4-chamber views using the in-built QLAB software in the CX50 echo machine. LV diastolic dysfunction will be assessed using mitral valve inflow velocities, mitral annular tissue doppler velocities, left atrial volume index, and tricuspid regurgitation jet velocity as per the guidelines.[62]

Scans will be performed by experienced echo technicians, two per centre, after a joint 2-day protocol-specific training session. Following this, the technical quality of scans will be checked by the senior cardiologist locally in each centre, and a set of 10 scans will be exchanged between centres for an independent quality assessment. Scans will be read by experienced cardiologists in each centre (AR in New Delhi and VST and AGA in Vellore). A random 10% of all scans recorded in the study will be exchanged and read by the other centre to assess inter-observer variability.

Carotid intimal media thickness (cIMT) measurement

Measurements of cIMT will be made bilaterally using high-resolution B-mode ultrasound with the same machine (Philips CX50 Compact Xtreme) and a 10-MHz linear array probe (Philips), and quantified using the CX50-IMT Philips Quantification application according to American Society for Echocardiography guidelines.[63] Each common carotid artery will be imaged in three different projections (anterior, lateral and posterior) proximal to the bifurcation. Measurements of IMT will be taken at a plaque-free zone in the far wall of the common carotid artery, captured at the end of diastole. The mean of three readings will be taken. The presence or absence of plaque, defined as a focal structure that encroaches upon the arterial lumen by at least 0.5 mm or is more than 50% of the surrounding intima-media thickness or has a thickness greater than or equal to 1.5 mm will also be recorded. Between measurer reproducibility in the LV parameters and cIMT will be assessed by the exchange of 10% of scans from each site.

For peer review only - http://bmjopen.bmj.com/site/about/guidelines.xhtml

Electrocardiogram

All participants will undergo a 12-lead ECG recording. ECGs will be reviewed by a cardiologist, and if there are changes suggestive of ischaemia (ST-depression and/or T wave inversion), detailed Minnesota Coding will be performed. We will use Minnesota codes 1-1 and 1-2, 4-1 and 4-2, 5-1 and 5-2 and 7-1 to indicate ischaemic heart disease.[64]

Body composition assessments

Body composition (total lean mass, fat mass and fat percent) will be assessed using bioelectrical impedance in both centres (Tanita BC-418 in NDBC and Bodystat-2500 in VBC). In VBC, a DXA examination will be additionally performed (Hologic Discovery) to obtain total and depot specific lean mass, fat mass and total fat percentage. DXA body composition analysis was assessed among NDBC participants in 2009[65] and will not be re-examined in IndEcho.

A copy of the full protocol can be obtained from the corresponding author.

Sample size and analysis

We will target the surviving cohort members (1,508 from NDBC and 2,156 from VBC) who participated in follow-ups between 1998 and 2002. From initial tracing in early 2016, a total of 3,500 individuals were re-contacted, among whom we expect approximately 1,250 in Delhi and 1,750 in Vellore to participate (total 3,000) in the current protocol.

Exposures in early life will include weight and length at birth; weight, height and BMI and independent conditional weight and height estimates of growth during infancy, childhood and adolescence, as previously described.[48] Because early growth measurements were measured at different time points and at slightly different ages in both cohorts, and owing to the skewness of BMI, the data will be transformed into SD scores using the LMS method.[66] The main analysis will use all available data at each timepoint; it will also be repeated using the sub-set of participants having data

For peer review only - http://bmjopen.bmj.com/site/about/guidelines.xhtml

for all time points (birth, infant, childhood and adolescent measurements). Exposures in young adult life will include lifestyle characteristics (diet, physical activity, smoking and alcohol consumption, SES, urban/rural residence and history of rural-urban migration (Vellore only)); anthropometry; lean and fat mass measured by DXA and bio-impedance; metabolic risk markers (plasma glucose, insulin, and lipids) and categorical abnormalities as defined above.

We will check the distributions of all variables and perform appropriate transformations. We will explore relationships between LV wall thickness and mass (as continuous variables) with current body size, and make appropriate adjustments, including the conventional adjustment for body surface area and height.[60] We will create categorical variables to represent LVH and ventricular dysfunction based on standard cut-offs for LV mass as described above.[61] We will examine associations of early life and young adult exposures with outcomes, using linear and logistic regression as appropriate, with and without adjustment for potential confounders and covariates (including age, sex, history of migration, current body size and composition). We will also use the conditional growth modelling approach to relate growth in BMI and height during discrete age periods with adult outcomes.[67 68] We will explore interactions between early life and young adult exposures and between early life exposures and current measures, using product terms. The role of young adult lifestyle and CVD risk markers as mediators of associations between early life exposures and outcomes will also be examined. In order to test the representativeness of the IndEcho participants and assess the risk of bias, we will compare their early growth variables (birth length, birth weight, and height, weight and BMI in infancy, childhood and early adulthood) with members of the original cohorts who did not participate as we have done previously (Table 2). Since all analyses in the IndEcho study will be 'internal' (within the sample of participants) bias will occur only if the associations between early growth and adult outcomes differ between those who participate in IndEcho and those who were not studied. We cannot think of a mechanism by which such differences could occur. We will examine the data from men and women, from Delhi and Vellore, and (in Vellore) among rural and urban participants separately, and assess heterogeneity of associations and scope for pooling.

For peer review only

Using tests at 5% significance, and a total sample size of 3,000, we will have 80% power to detect an association of 0.05 SDs of a continuous outcome (eg LV mass) per SD change in a continuous predictor (eg birth weight or conditional weight gain in infancy). For the Vellore cohort alone (based on N=1,750) this figure is 0.07 SDs and for the Delhi cohort alone (based on N=1,250) it is 0.08 SDs. These figures compare favorably with the association shown between infant weight and adult LV mass in the Hertfordshire cohort (0.17 SD per SD change in infant weight).[26] For a binary outcome such as LV diastolic dysfunction (Grade 1 & 2), assuming 10% prevalence and a test at 5% significance level, we will have 80% power to detect an association of 0.17 log odds per SD change in a continuous predictor, equivalent to an odds ratio of 1.19 (Vellore 1.25, Delhi 1.30).

DISCUSSION

To the best of our knowledge, IndEcho will be the first study among South Asians to provide data linking birth size and childhood growth, as well as prior measures of CVD risk markers and lifestyle factors such as physical activity and smoking in young adulthood, with cardiac structure and function and in middle age. It will provide an opportunity to study interactions between early life, young adult and concurrent risk factors in relation to myocardial outcomes.

The main strengths of the study are that it includes participants from two large Indian birth cohorts that have serial childhood growth data, measured prospectively by trained research staff, and risk factors for CVD (including lifestyle risk factors) assessed 16-20 years ago. Harmonised methods will be used in both cohorts. The participants represent north and south Indian populations, different socioeconomic strata, and rural and urban settings. Because we have serial measures of socio-economic status, collected using identical methods, IndEcho provides an opportunity to assess the impact of economic transition on cardiac health in Indians. At this age we will detect sub-clinical cardiac disease in an apparently healthy population where obesity, hypertension and T2DM are escalating. In 1998-2002 at age 26-32 years, the prevalence of obesity (BMI $\geq$30 kg/m^2), hypertension (systolic $\geq$140 mmHg or diastolic $\geq$90 mmHg or on treatment for hypertension) and diabetes (WHO criteria) was 11%, 8% and 4% respectively in NDBC[29] and 2%, 3% and 3% in VBC.[31]

For peer review only - http://bmjopen.bmj.com/site/about/guidelines.xhtml

Approximately 5 years later, at 34-39 years, the prevalence had more than doubled in NDBC (23%, 26% and 9%).[45] In the 2013-14 VBC follow-up, the equivalent data (16%, 20% and 17%) showed a more than 5-fold increase in prevalence in just over a decade (unpublished data).

Like all birth cohorts of this age, an important limitation is loss to follow-up, mainly due to deaths in childhood, and migration out of the original study area. However, current cohort members were similar in early life to the rest of the original cohorts (Table 2).[29 48] In order to create bias, the relationship between the predictors (eg. birth weight, or young adult diabetes) and outcomes (eg. LV mass) would have to differ between those studied and not studied. Another limitation is that, because this is a field-based study, echocardiography will be used, rather than cardiac MRI, which is the current gold standard method for measuring myocardial structure

The findings of the IndEcho study will help plan effective strategies in early life and/or young adulthood to prevent adult CVD in India and migrant South Asian populations. We expect that it will identify periods during early life when nutritional interventions may prevent later cardiac disease. As the prospective follow-up continues in future, and as cohort members start to develop cardiac disease, we will also be able to define the value of LV measurements for predicting cardiovascular morbidity and mortality, which will be the first such data from India.

ETHICS AND DISSEMINATION

IndEcho does not involve any invasive or risky procedures for participants. Participant information sheets and consent forms are made available in English and local languages, describing the purpose of the study and the various procedures in lay terms. Participation is voluntary and participants provide written informed consent before enrollment. The study has been approved by the respective ethics committees of the participating institutions and is registered as ISRCTN13432279. Findings from the study will be disseminated through presentations at both international and national conferences and results generated will be published in international peer-reviewed journals.

For peer review only - http://bmjopen.bmj.com/site/about/guidelines.xhtml

Author affiliations

The study will involve investigators based in India (New Delhi and Vellore) and the UK. The participating institutions in New Delhi, India include Sunder Lal Jain Hospital (Santosh K Bhargava, Bhaskar Singh), Sitaram Bhartia Institute of Science and Research Institute (HPS Sachdev, Sikha Sinha), Centre for Chronic Disease Control & Public Health Foundation of India (Prabhakaran Dorairaj), All-India Institute of Medical Sciences (Ambuj Roy, Nikhil Tandon, Lakshmy Ramakrishnan). The representatives of the VBC from the Christian Medical College, Vellore include from the Departments of Biostatistics (Antonisamy, Prasanna S Premkumar, Sneha FX Princy), Endocrinology (Nihal Jacob Thomas, Thomas Paul, Prrathepa Asaithambi, Kanhaiya Agrawal) Clinical Biochemistry (Finney S Geethanjali), and Cardiology (Anoop George Alexander and Viji Thomson Samuel). The two UK-based institutions include The Oxford Centre for Diabetes, Endocrinology and Metabolism, Oxford University, Oxford, UK (Fredrik Karpe) and MRC Lifecourse Epidemiology Unit, University of Southampton, UK (Senthil K Vasan, Clive Osmond and Caroline Fall).

Acknowledgements

We thank the members of the NBDC and VBC, and their families, for their participation in research over more than 4 decades. We acknowledge the IndEcho field teams in Delhi and Vellore, which include administrative staff, field workers and supervisors, research officers, research nurses, phlebotomists, DXA and echo technicians, data management teams and data entry operators.

ADDITIONAL INFORMATION

Authors' contributors

SKV[1], AR[2], VTS[3], BA[4], SKB[5], AAG[6], CO[7], FK[8], HS[9], LR[10], NT[11], NJT[12], TVP[13], DP[14] and CHD[15] conceived the study and wrote the study protocol. BS[16], FSG[17], KA[18], PSP[19], PA[20], SFXP[21] and SS[22] will contribute significantly to the acquisition of the data. CO[7], SKV[1], FK[8], DP[14], SKB[5], HS[9], AR[2], VTS[3], AAG[6], BA[4], PSP[19], SFXP[21] and CHDF[15] significantly contributed to the planning of analyses of the data. SKV[1] and CHDF[15] drafted the first version of the manuscript and all authors revised the

For peer review only

manuscript critically for important intellectual content. All authors reviewed and approved the final manuscript and agree to be accountable for all aspects of the work

Funding

The original cohort studies were supported by the National Center for Health Statistics, USA and the Indian Council of Medical Research. The two earlier follow-up studies in young adult life were supported by the British Heart Foundation. The IndEcho study is supported by British Heart Foundation Clinical Research Grant, No. CRM:0022324. Professor Fall's work on the study is supported by the UK Medical Research Council (MRC) and the UK Department for International Development (DFID) under the MRC/DFID Concordat.

Competing interests: None declared.

Ethics approval

The study has been approved by the research ethics committees of Sunder Lal Jain Hospital, New Delhi (13th August 2015; SLJ/IEC/1); Sitaram Bhartia Institute of Science and Research, New Delhi (23rd October 2015; IEC/SBSR/2015/1); Centre for Chronic Disease Control, New Delhi (no.50/7/TF-CVD/15-NCD-II); All-India Institute of Medical Sciences, New Delhi (21st October 2015, IEC/NP-410/09.10.2015); Christian Medical College, Vellore (22nd July 2015; IRB 9548[OBSERV]) and the Faculty of Medicine, University of Southampton, UK (11th April 2016; RE 18694).

Provenance and peer review Not commissioned; externally peer reviewed.

Data sharing statement Data collection for this study is ongoing.

For peer review only

References

1. Mortality GBD, Causes of Death C. Global, regional, and national life expectancy, all-cause mortality, and cause-specific mortality for 249 causes of death, 1980-2015: a systematic analysis for the Global Burden of Disease Study 2015. *Lancet* 2016;388(10053):1459-544.
2. Disease GBD, Injury I, Prevalence C. Global, regional, and national incidence, prevalence, and years lived with disability for 310 diseases and injuries, 1990-2015: a systematic analysis for the Global Burden of Disease Study 2015. *Lancet* 2016;388(10053):1545-602.
3. Roth GA, Johnson C, Abajobir A, et al. Global, Regional, and National Burden of Cardiovascular Diseases for 10 Causes, 1990 to 2015. *J Am Coll Cardiol* 2017;70(1):1-25.
4. McKeigue PM, Miller GJ, Marmot MG. Coronary heart disease in south Asians overseas: a review. *J Clin Epidemiol* 1989;42(7):597-609.
5. Wild S, McKeigue P. Cross sectional analysis of mortality by country of birth in England and Wales, 1970-92. *BMJ* 1997;314(7082):705-10.
6. Yusuf S, Reddy S, Ounpuu S, et al. Global burden of cardiovascular diseases: Part II: variations in cardiovascular disease by specific ethnic groups and geographic regions and prevention strategies. *Circulation* 2001;104(23):2855-64.
7. Lim SS, Vos T, Flaxman AD, et al. A comparative risk assessment of burden of disease and injury attributable to 67 risk factors and risk factor clusters in 21 regions, 1990-2010: a systematic analysis for the Global Burden of Disease Study 2010. *Lancet* 2012;380(9859):2224-60.
8. Collaboration NCDRF. Worldwide trends in diabetes since 1980: a pooled analysis of 751 population-based studies with 4.4 million participants. *Lancet* 2016;387(10027):1513-30.
9. Levy D, Garrison RJ, Savage DD, et al. Left ventricular mass and incidence of coronary heart disease in an elderly cohort. The Framingham Heart Study. *Ann Intern Med* 1989;110(2):101-7.
10. Abel ED, Litwin SE, Sweeney G. Cardiac remodeling in obesity. *Physiol Rev* 2008;88(2):389-419.
11. Boudina S, Abel ED. Diabetic cardiomyopathy revisited. *Circulation* 2007;115(25):3213-23.
12. Lauer MS, Anderson KM, Kannel WB, et al. The impact of obesity on left ventricular mass and geometry. The Framingham Heart Study. *JAMA* 1991;266(2):231-6.
13. Desai CS, Colangelo LA, Liu K, et al. Prevalence, prospective risk markers, and prognosis associated with the presence of left ventricular diastolic dysfunction in young adults: the coronary artery risk development in young adults study. *Am J Epidemiol* 2013;177(1):20-32.
14. Schirmer H, Lunde P, Rasmussen K. Mitral flow derived Doppler indices of left ventricular diastolic function in a general population; the Tromso study. *Eur Heart J* 2000;21(16):1376-86.
15. Tillin T, Hughes AD, Mayet J, et al. The relationship between metabolic risk factors and incident cardiovascular disease in Europeans, South Asians, and African Caribbeans: SABRE (Southall and Brent Revisited) -- a prospective population-based study. *J Am Coll Cardiol* 2013;61(17):1777-86.

For peer review only

16. Prabhakaran D, Jeemon P, Roy A. Cardiovascular Diseases in India: Current Epidemiology and Future Directions. *Circulation* 2016;133(16):1605-20.
17. Bellary S, O'Hare JP, Raymond NT, et al. Premature cardiovascular events and mortality in south Asians with type 2 diabetes in the United Kingdom Asian Diabetes Study - effect of ethnicity on risk. *Curr Med Res Opin* 2010;26(8):1873-9.
18. Bilen O, Kamal A, Virani SS. Lipoprotein abnormalities in South Asians and its association with cardiovascular disease: Current state and future directions. *World J Cardiol* 2016;8(3):247-57.
19. McKeigue PM, Shah B, Marmot MG. Relation of central obesity and insulin resistance with high diabetes prevalence and cardiovascular risk in South Asians. *Lancet* 1991;337(8738):382-6.
20. Raji A, Seely EW, Arky RA, et al. Body fat distribution and insulin resistance in healthy Asian Indians and Caucasians. *J Clin Endocrinol Metab* 2001;86(11):5366-71.
21. Yajnik CS, Fall CH, Coyaji KJ, et al. Neonatal anthropometry: the thin-fat Indian baby. The Pune Maternal Nutrition Study. *Int J Obes Relat Metab Disord* 2003;27(2):173-80.
22. Yajnik CS, Lubree HG, Rege SS, et al. Adiposity and hyperinsulinemia in Indians are present at birth. *J Clin Endocrinol Metab* 2002;87(12):5575-80.
23. Seaton SE, Yadav KD, Field DJ, et al. Birthweight centile charts for South Asian infants born in the UK. *Neonatology* 2011;100(4):398-403.
24. Joglekar CV, Fall CH, Deshpande VU, et al. Newborn size, infant and childhood growth, and body composition and cardiovascular disease risk factors at the age of 6 years: the Pune Maternal Nutrition Study. *Int J Obes (Lond)* 2007;31(10):1534-44.
25. Curhan GC, Willett WC, Rimm EB, et al. Birth weight and adult hypertension, diabetes mellitus, and obesity in US men. *Circulation* 1996;94(12):3246-50.
26. Osmond C, Barker DJ, Winter PD, et al. Early growth and death from cardiovascular disease in women. *BMJ* 1993;307(6918):1519-24.
27. Barker DJ. Fetal programming of coronary heart disease. *Trends Endocrinol Metab* 2002;13(9):364-8.
28. Stein CE, Fall CH, Kumaran K, et al. Fetal growth and coronary heart disease in south India. *Lancet* 1996;348(9037):1269-73.
29. Bhargava SK, Sachdev HS, Fall CH, et al. Relation of serial changes in childhood body-mass index to impaired glucose tolerance in young adulthood. *N Engl J Med* 2004;350(9):865-75.
30. Bavdekar A, Yajnik CS, Fall CH, et al. Insulin resistance syndrome in 8-year-old Indian children: small at birth, big at 8 years, or both? *Diabetes* 1999;48(12):2422-9.
31. Raghupathy P, Antonisamy B, Geethanjali FS, et al. Glucose tolerance, insulin resistance and insulin secretion in young south Indian adults: Relationships to parental size, neonatal size and childhood body mass index. *Diabetes Res Clin Pract* 2010;87(2):283-92.
32. Crispi F, Bijnens B, Figueras F, et al. Fetal growth restriction results in remodeled and less efficient hearts in children. *Circulation* 2010;121(22):2427-36.
33. Crispi F, Figueras F, Cruz-Lemini M, et al. Cardiovascular programming in children born small for gestational age and relationship with prenatal signs of severity. *Am J Obstet Gynecol* 2012;207(2):121 e1-9.

For peer review only

34. Tsyvian P, Malkin K, Artemieva O, et al. Cardiac ventricular performance in the appropriate- for-gestational age and small-for-gestational age fetus: relation to regional cardiac non-uniformity and peripheral resistance. *Ultrasound Obstet Gynecol* 2002;20(1):35-41.
35. Thornburg KL, Louey S. Fetal roots of cardiac disease. *Heart* 2005;91(7):867-8.
36. Hietalampi H, Pahkala K, Jokinen E, et al. Left ventricular mass and geometry in adolescence: early childhood determinants. *Hypertension* 2012;60(5):1266-72.
37. Vijayakumar M, Fall CH, Osmond C, et al. Birth weight, weight at one year, and left ventricular mass in adult life. *Br Heart J* 1995;73(4):363-7.
38. Zureik M, Bonithon-Kopp C, Lecomte E, et al. Weights at birth and in early infancy, systolic pressure, and left ventricular structure in subjects aged 8 to 24 years. *Hypertension* 1996;27(3 Pt 1):339-45.
39. Li X, Li S, Ulusoy E, et al. Childhood adiposity as a predictor of cardiac mass in adulthood: the Bogalusa Heart Study. *Circulation* 2004;110(22):3488-92.
40. Sivanandam S, Sinaiko AR, Jacobs DR, Jr., et al. Relation of increase in adiposity to increase in left ventricular mass from childhood to young adulthood. *Am J Cardiol* 2006;98(3):411-5.
41. Blackledge HM, Newton J, Squire IB. Prognosis for South Asian and white patients newly admitted to hospital with heart failure in the United Kingdom: historical cohort study. *BMJ* 2003;327(7414):526-31.
42. Chahal NS, Lim TK, Jain P, et al. Ethnicity-related differences in left ventricular function, structure and geometry: a population study of UK Indian Asian and European white subjects. *Heart* 2010;96(6):466-71.
43. Kumaran K, Fall CH, Martyn CN, et al. Left ventricular mass and arterial compliance: relation to coronary heart disease and its risk factors in South Indian adults. *Int J Cardiol* 2002;83(1):1-9.
44. Kumaran K, Fall CH, Martyn CN, et al. Blood pressure, arterial compliance, and left ventricular mass: no relation to small size at birth in south Indian adults. *Heart* 2000;83(3):272-7.
45. Huffman MD, Prabhakaran D, Osmond C, et al. Incidence of cardiovascular risk factors in an Indian urban cohort results from the New Delhi birth cohort. *J Am Coll Cardiol* 2011;57(17):1765-74.
46. Antonisamy B, Raghupathy P, Christopher S, et al. Cohort Profile: the 1969-73 Vellore birth cohort study in South India. Int J Epidemiol 2009;38(3):663-9.
47. Khalil A, Huffman MD, Prabhakaran D, et al. Predictors of carotid intima-media thickness and carotid plaque in young Indian adults: the New Delhi birth cohort. *Int J Cardiol* 2013;167(4):1322-8.
48. Antonisamy B, Vasan SK, Geethanjali FS, et al. Weight Gain and Height Growth during Infancy, Childhood, and Adolescence as Predictors of Adult Cardiovascular Risk. *J Pediatr* 2017;180:53-61 e3.
49. Raghupathy P, Antonisamy B, Fall CH, et al. High prevalence of glucose intolerance even among young adults in south India. *Diabetes Res Clin Pract* 2007;77(2):269-79.
50. National Institute of Nutrition. National Nutrition Monitoring Bureau Report of Repeat Surveys (1998-90). In: Indian Council of Medical Research. Hyderabad I, ed., 1991.
51. Bassani DG, Corsi DJ, Gaffey MF, et al. Local distributions of wealth to describe health inequalities in India: a new approach for analyzing nationally

For peer review only

representative household survey data, 1992-2008. *PLoS One* 2014;9(10):e110694.
52. Ramesh Masthi NR, Gangaboraiah, Kulkarni P. An exploratory study on socio economic status scales in a rural and urban setting. *J Family Med Prim Care* 2013;2(1):69-73.
53. International Institute for Population Sciences and Macro International. National Family Health Survey (NFHS—2): International Institute of Population Sciences, Mumbai., 1998-99.
54. Bull FC, Maslin TS, Armstrong T. Global physical activity questionnaire (GPAQ): nine country reliability and validity study. *J Phys Act Health* 2009;6(6):790-804.
55. Anjana RM, Pradeepa R, Das AK, et al. Physical activity and inactivity patterns in India - results from the ICMR-INDIAB study (Phase-1) [ICMR-INDIAB-5]. *Int J Behav Nutr Phys Act* 2014;11(1):26.
56. Rani M, Bonu S, Jha P, et al. Tobacco use in India: prevalence and predictors of smoking and chewing in a national cross sectional household survey. *Tob Control* 2003;12(4):e4.
57. World Health Organization: Definition, Diagnosis and Classification of Diabetes Mellitus and its. Complications: Report of a WHO Consultation., 1999.
58. James PA, Oparil S, Carter BL, et al. 2014 evidence-based guideline for the management of high blood pressure in adults: report from the panel members appointed to the Eighth Joint National Committee (JNC 8). *JAMA* 2014;311(5):507-20.
59. Alberti KGMM, Zimmet P, Shaw J. The metabolic syndrome - a new worldwide definition. *Lancet* 2005;366(9491):1059-62.
60. Lang RM, Badano LP, Mor-Avi V, et al. Recommendations for cardiac chamber quantification by echocardiography in adults: an update from the American Society of Echocardiography and the European Association of Cardiovascular Imaging. *J Am Soc Echocardiogr* 2015;28(1):1-39 e14.
61. Lang RM, Bierig M, Devereux RB, et al. Recommendations for chamber quantification. *Eur J Echocardiogr* 2006;7(2):79-108.
62. Nagueh SF, Smiseth OA, Appleton CP, et al. Recommendations for the Evaluation of Left Ventricular Diastolic Function by Echocardiography: An Update from the American Society of Echocardiography and the European Association of Cardiovascular Imaging. *J Am Soc Echocardiogr* 2016;29(4):277-314.
63. Stein JH, Korcarz CE, Hurst RT, et al. Use of carotid ultrasound to identify subclinical vascular disease and evaluate cardiovascular disease risk: a consensus statement from the American Society of Echocardiography Carotid Intima-Media Thickness Task Force. Endorsed by the Society for Vascular Medicine. *J Am Soc Echocardiogr* 2008;21(2):93-111; quiz 89-90.
64. Prineas RJ CR, Blackburn H. The Minnesota code manual of electrocardiographic findings: standards and procedures for measurement and classification. Boston, Wright 1982.
65. Tandon N, Fall CH, Osmond C, et al. Growth from birth to adulthood and peak bone mass and density data from the New Delhi Birth Cohort. *Osteoporos Int* 2012;23(10):2447-59.
66. Cole TJ, Green PJ. Smoothing reference centile curves: the LMS method and penalized likelihood. *Stat Med* 1992;11(10):1305-19.

For peer review only

67. Osmond C, Fall CHD. Conditional Growth Models: an Exposition and some Extensions. The Association Between Early Growth and a Later Outcome., Disease Modelling and Public Health. 2017.
68. Adair LS, Fall CH, Osmond C, et al. Associations of linear growth and relative weight gain during early life with adult health and human capital in countries of low and middle income: findings from five birth cohort studies. *Lancet* 2013;382(9891):525-34.

For peer review only

Table 1 IndEcho study procedures and platforms used

Study-related procedures	Methods /platforms used
Questionnaire assessments	
Diet	Food Frequency Questionnaire (FFQ)
Physical activity	General Physical Activity Questionnaire (GPAQ)
Socioeconomic status	NFHS Standard of Living Index
Smoking	NFHS-2 Household Questionnaire
Alcohol consumption	NFHS-2 Household Questionnaire
Anthropometry	
Height	Stadiometer
Weight	Digital weighing scales
Waist circumference	Non-stretchable tape
Hip circumference	Non-stretchable tape
Blood pressure	Omron M3
Biochemistry	
Glucose - fasting	Enzymatic method (autoanalyser)
Glucose – 120 min	Enzymatic method (autoanalyser)
Insulin - fasting	Vellore: Radio-fluorimetric method<br>Delhi: Chemiluminescence immunoassay
Cholesterol	CHOD-PAP Enzymatic colorimetric method
Triglycerides	GPO-PAP Enzymatic colorimetric method
HDL-cholesterol	Direct – two step enzymatic
LDL-cholesterol	Direct – Enzymatic colorimetric method
Urinary ACR	Jaffe Method
Skin fold thickness	John Bull/Harpenden skinfold calliper
Bioimpedence	Tanita BC-418/ Bodystat 2500
Hand Grip	JAMAR dynamometer
DXA*	Hologic Discovery
Electrocardiogram (ECG)	
Echocardiography	Philips CX50 Compact Xtreme system
CIMT#	Philips CX50 Compact Xtreme system

FFQ developed by the National Institute of Nutrition, Hyderabad[51]
SES Questionnaire developed by the National Family Health Survey (NFHS-2), 1998-99[52]
GPAQ: Global Physical Activity Questionnaire developed by WHO[53]
*DXA- Dual Energy X-ray absorptiometry
#CIMT- Carotid Intimal Media thickness

For peer review only

Table 2: Comparison of early growth measurements of studied and not-studied participants during Phase 1 adult follow up

Measurement	New Delhi Birth Cohort				Vellore Birth Cohort			
	Male		Female		Male		Female	
	Studied as adult		Studied as adult		Studied as adult		Studied as adult	
	Yes	No	Yes	No	Yes	No	Yes	No
**Birth**								
n	808	2642	569	2497	1159	1712	1058	1741
Weight (kg)	2.89 ± 0.44	2.86 ± 0.46	2.79 ± 0.38	2.78 ± 0.44	2.85 ± 0.53	2.83 ± 0.61	2.78 ± 0.5	2.76 ± 0.54
Length (cm)	48.8 ± 2.2	48.7 ± 2.4	48.3 ± 1.9	48.1 ± 2.2	48.3 ± 3.0	47.8 ± 4.6	47.8 ± 3.0	47.3 ± 4.3
Ponderal Index (kg/m^3)	24.8 ± 2.6	24.7 ± 2.9	24.7 ± 2.5	24.9 ± 2.9	25.7 ± 7.6	25.6 ± 7.3	25.7 ± 6.6	25.9 ± 7.3
Gestation (wk)	39.2 ± 2.2	39.2 ± 2.3	39.6 ± 2.0	39.6 ± 2.2	38.2 ± 2.8	38.1 ± 2.9	38.3 ± 2.8	38.4 ± 2.8
**Infancy (3 months)**								
n	660	2140	503	2105	845	1011	791	1108
Weight (kg)	5.50 ± 0.7	5.46 ± 0.8	4.96 ± 0.7	4.99 ± 0.7	4.28 ± 0.8	4.17 ± 0.9	4.04 ± 0.7	3.91 ± 0.8
Height (cm)	59.6 ± 2.4	59.5 ± 2.7	58.0 ± 2.4	58.1 ± 2.5	55.2 ± 3.1	55.1 ± 3.6	54.3 ± 2.9	54.2 ± 3.2
BMI (kg/m^2)	15.4 ± 1.5	15.4 ± 1.6	14.7 ± 1.6	14.7 ± 1.6	14.1 ± 1.9	13.6 ± 2.2	13.6 ± 1.7	13.12 ± 1.92
**Childhood (6 years)**								
n	837	1092	608	1219	1001	601	933	645
Weight (kg)	17.2 ± 2.2	17.3 ± 2.3	16.4 ± 2.0	16.5 ± 2.2	15.8 ± 4.4	15.5 ± 4.4	15.4 ± 4.3	15.4 ± 4.2
Height (cm)	108.4 ± 5.2	108.9 ± 5.5	107.0 ± 5.1	107.2 ± 5.5	102.4 ± 18.7	100.1 ± 26.4	100.6 ± 19.7	100.6 ± 23.5
BMI (kg/m^2)	14.6 ± 1.1	14.5 ± 1.1	14.3 ± 1.1	14.3 ± 1.2	15.4 ± 4.4	15.3 ± 4.1	15.6 ± 4.8	15.2 ± 4.11
**Adolescence (15 years)**								
n	616	435	481	592	775	467	727	508
Weight (kg)	44.6 ± 9.2	45.8 ± 9.6	44.6 ± 7.8	44.2 ± 7.5	27.7 ± 5.8	27.5 ± 6.3	28 ± 5.37	28.5 ± 6.1
Height (cm)	159.2 ± 8.7	160.2 ± 8.4	153.2 ± 5.7	153.3 ± 6.2	136.5 ± 9.9	137.1 ± 13.6	135.3 ± 8.0	136.5 ± 9.9
BMI (kg/m^2)	17.5 ± 2.5	17.7 ± 2.7	18.9 ± 3.0	18.8 ± 2.7	14.7 ± 1.4	14.6 ± 1.6	15.2 ± 1.9	15.2 ± 1.8
**Adulthood**								
n	886	-	640	-	1159	-	1053	

For peer review only - http://bmjopen.bmj.com/site/about/guidelines.xhtml

Age (years)	29.2 ± 1.3	-	29.2 ± 1.4	-	27.9 ± 1.1	-	28.3 ± 1.2	-
Weight (kg)	71.8 ± 14.0	-	59.2 ± 13.4	-	57.4 ± 11.4	-	49.3 ± 10.6	-
Height (cm)	169.7 ± 6.4	-	154.9 ±5.7	-	166.4 ± 6.7	-	153.8 ± 6.0	-
BMI (kg/m^2)	24.9 ± 4.3	-	24.6 ± 5.1	-	20.7 ± 3.5	-	20.8 ± 4.1	-

For peer review only

For peer review only - http://bmjopen.bmj.com/site/about/guidelines.xhtml

Figure legends

Figure 1

Title: BMI SD scores from birth to adulthood for participants in each cohort who developed impaired glucose tolerance (IGT) or diabetes (N=219/1562 in NDBC and 424/2218 in VBC) in adult life relative to the whole cohort (dashed zero line)

Legend: No legend

Figure 2

Title: Pathways to altered left ventricular structure and function that will be investigated in IndEcho

Legend: Factors contributing to left ventricular hypertrophy. Intra-uterine under-nutrition alters the fetal circulation, which may have direct and persistent effects on ventricular structure. Intra-uterine and infant under-nutrition impairs the development of key metabolic tissues (muscle, pancreas, liver, blood vessels and kidneys) during critical periods of growth, and increases adipose tissue deposition, leading to the 'thin-fat' phenotype, and later obesity, T2DM, dyslipidaemia, hypertension and chronic renal disease, which adversely impact on LV size and function.

Figure 3

Title: Flow chart of various stages of follow-up of the New Delhi and Vellore Birth Cohorts and measurements recorded at each stage.

Legend: BMI: Body mass index; WC: Waist circumference; HC: Hip circumference; SES: Socio-economic status; PA: Physical activity; OGTT: Oral glucose tolerance test; ECG: Electrocardiogram; DXA: Dual energy X-ray absorptiometry

For peer review only

For peer review only - http://bmjopen.bmj.com/site/about/guidelines.xhtml

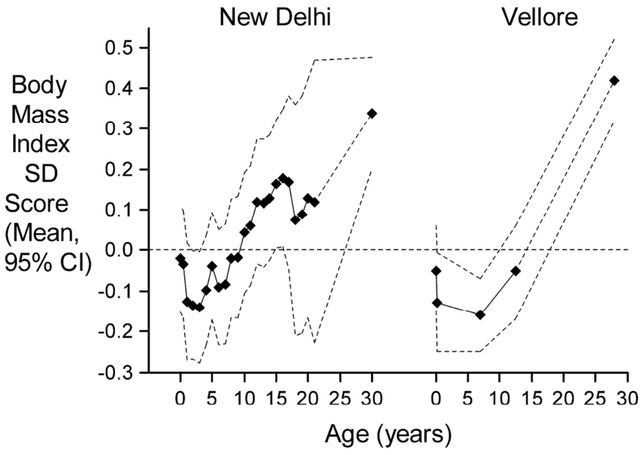

BMI SD scores from birth to adulthood for participants in each cohort who developed impaired glucose tolerance (IGT) or diabetes (N=219/1562 in NDBC and 424/2218 in VBC) in adult life relative to the whole cohort (dashed zero line)

190x107mm (300 x 300 DPI)

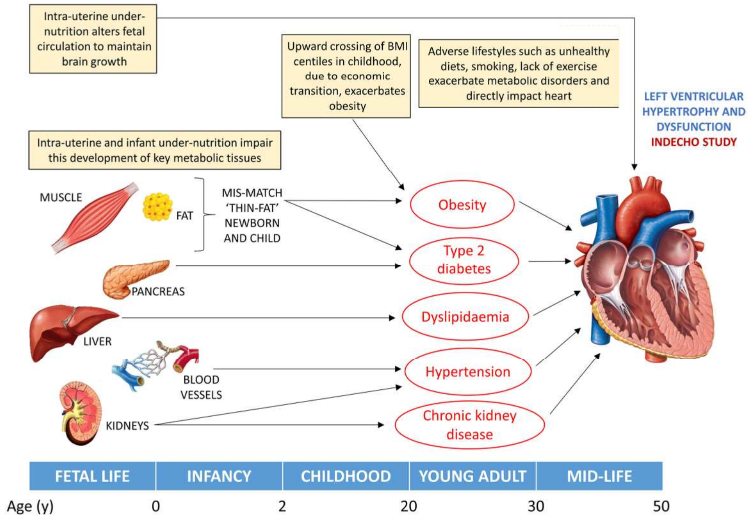

Pathways to altered left ventricular structure and function that will be investigated in IndEcho / Legend: Factors contributing to left ventricular hypertrophy. Intra-uterine under-nutrition alters the fetal circulation, which may have direct and persistent effects on ventricular structure. Intra-uterine and infant under-nutrition impairs the development of key metabolic tissues (muscle, pancreas, liver, blood vessels and kidneys) during critical periods of growth, and increases adipose tissue deposition, leading to the 'thin-fat' phenotype, and later obesity, T2DM, dyslipidaemia, hypertension and chronic renal disease, which adversely impact on LV size and function.

190x107mm (300 x 300 DPI)

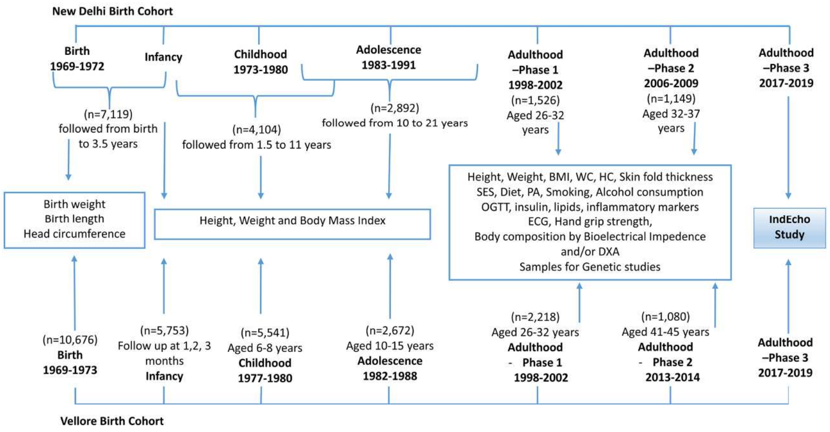

Flow chart of various stages of follow-up of the New Delhi and Vellore Birth Cohorts and measurements recorded at each stage./ Legend: BMI: Body mass index; WC: Waist circumference; HC: Hip circumference; SES: Socio-economic status; PA: Physical activity; OGTT: Oral glucose tolerance test; ECG: Electrocardiogram; DXA: Dual energy X-ray absorptiometry.

190x107mm (300 x 300 DPI)

For peer review only

Supplementary table 1: Total number of participants in Phase 1 and 2 of NDBC and VBC and reasons for lost to follow-up between both adult phases.

	NDBC	VBC
**Phase-1**	(1998-2002)	(1998-2002)
Age (years)	29.3 ± 1.0	28.3 ± 1.2
Total (N)	1,526	2,218
Men (N)	886	1,161
Women (N)	640	1,057
**Phase-2**	(2006-2009)	(2013-2014)
Age (years)	36.1 ± 1.1	41.6 ± 1.0
Total (N)	1,100	1,080
Men (N)	665	581
Women (N)	484	499
**Numbers lost to follow-up between Phase 1 and Phase 2 and reasons**	426	1,138
Known deaths (N)	18	62
Known migration (N)	88	50
Unwilling to participate (N)	266	49
Not traceable (N)	54	21
Funding restrictions (N)	0	956

For peer review only

Supplementary table 2: Co-variates that will be studied to assess lifestyle and behavior in the IndEcho participants

Exposure variable	Questionnaire	Variables included in the questionnaire
Socioeconomic status (SES)[51-53]	The Government of India National Family Health Survey (NFHS-2) questionnaire and Standard of Living Index (SLI – IIPS,2000) questionnaire that contains 11 items to measure SES for both rural and urban populations of the entire country	Includes information on:<br>(i) Education<br>(ii) Occupation<br>(iii) Household characteristics (family type, number of persons)<br>(iv) SLI is a summary household measure composed of 11 items, including (a) house type (b) source of lighting (c) toilet facilities (d) main fuel for cooking (e) source of drinking water (f) separate room for cooking (g) ownership of house (h) ownership of agricultural land (i) ownership of irrigated land (j) ownership of livestock (k) ownership of durable goods
Physical activity (PA)[54,55]	Global Physical Activity Questionnaire (GPAQ)	Includes questions related to three domains<br>(i) Activity at work and in leisure time (vigorous- and moderate-intensity activity)<br>(ii) Travel to and from places<br>(iii) Recreational activities (vigorous and moderate recreation) and sitting behavior
Smoking and alcohol consumption[56]	NFHS-2 Questionnaire	Duration and quantity/type of tobacco consumed, smoking habits and alcohol intake

For peer review only - http://bmjopen.bmj.com/site/about/guidelines.xhtml
